# Real-Time WBAN Monitoring: An Adaptive Framework for Selective Signal Restoration and Physiological Trend Prediction

**DOI:** 10.3390/s26051684

**Published:** 2026-03-06

**Authors:** Fatimah Alghamdi, Fuad Bajaber

**Affiliations:** Department of Information Technology, Faculty of Computing and Information Technology, King Abdulaziz University, Jeddah 21589, Saudi Arabia; fbajaber@kau.edu.sa

**Keywords:** wireless body area networks (WBANs), streaming data, sliding-window technique, extended Kalman filtering, data imputation, signal smoothing, clipping, vital sign monitoring, intelligent health decision support

## Abstract

Wireless Body Area Networks (WBANs) enable real-time health monitoring essential for timely clinical intervention, yet their performance is frequently hindered by sensor degradation, noise interference, and strict low-latency constraints in resource-limited environments. Conventional preprocessing approaches indiscriminately reprocess all incoming data, including uncorrupted samples, thereby increasing computational overhead, introducing latency, and potentially distorting valid physiological trends. This study introduces a unified real-time monitoring framework tailored for WBAN systems. The key contributions include: (1) an adaptively gated multi-stage preprocessing pipeline that selectively restores corrupted samples while preserving clean data, (2) an overlap-aware sliding-window mechanism enabling low-latency operation, and (3) a clinically informed risk assessment strategy for early-warning support. By avoiding unnecessary modification of intact signals, the framework maintains physiological integrity while substantially improving reconstruction and predictive reliability. Across multiple vital signs, the proposed approach achieves substantial reconstruction gains, with Mean Squared Error (MSE) reductions ranging from 53% to 67% under strong degradation conditions. An adaptive ARIMA-based forecasting layer captures short-term physiological dynamics with directional accuracies of approximately 65–70% for one-step (10 s) ahead prediction. Early-warning behavior is intentionally conservative, prioritizing false alarm suppression over aggressive alerting. Per-signal evaluation reveals high sensitivity for blood pressure signals, whereas glucose and certain high-variability modalities exhibit conservative sensitivity under modality-specific thresholds. Importantly, the aggregated multi-modal risk decision achieves strong overall system-level performance, with sensitivity and specificity of 0.89 and 0.92, respectively. Overall, the proposed framework establishes a robust, low-latency, and computationally efficient foundation for dependable physiological monitoring in WBAN environments, leveraging selective processing to optimize both resource utilization and clinical reliability.

## 1. Introduction

Real-world WBAN deployments commonly experience packet loss rates between 5 and 30%, motion-induced impulse artifacts, nonlinear sensor saturation, and quantization errors. Under such conditions, uniform preprocessing of all samples leads to two critical issues: (i) unnecessary computational overhead proportional to window length *N* and (ii) distortion of already clean physiological segments. This motivates a selective restoration paradigm in which signal enhancement is applied only to statistically corrupted regions. The escalating global burden of chronic diseases and the imperative for proactive healthcare interventions underscore the critical need for advanced real-time physiological monitoring systems [1]. Wireless Body Area Networks (WBANs) have emerged as a pivotal technology, offering continuous, non-invasive data streams from wearable sensors for applications ranging from remote patient monitoring to acute clinical interventions [2]. However, the full potential of WBANs is severely hampered by the inherent challenges of pervasive physiological data degradation (e.g., missing values, nonlinear noise, motion artifacts), stringent demands for low-latency and reliable trend prediction, and the fundamental constraints of resource-limited environments, which collectively undermine the ability of current monitoring systems to provide high-fidelity, actionable insights essential for timely clinical decision-making [3,4].

Conventional approaches typically address data quality and predictive modeling in isolation, leading to fragmented solutions that inherently introduce delays and suboptimal performance, thereby limiting their clinical utility [5]. Furthermore, a common oversight in many existing preprocessing pipelines is the indiscriminate application of complex restoration techniques to all incoming data, regardless of their degradation level [6]. This excessive reprocessing, even of uncorrupted or minimally corrupted segments, can be counterproductive, introducing artificial artifacts, increasing computational burden, and ultimately leading to suboptimal or even erroneous downstream analyses, which constitutes a distinct research challenge in the preprocessing field [7]. For instance, while individual techniques such as various interpolation methods, noise filtering algorithms, or time-series forecasting models (e.g., ARIMA) have been extensively explored, their application in isolation proves insufficient for the complex, multi-faceted challenges of WBANs [8,9]. A simple imputation [10] method might reconstruct missing data but can inadvertently distort underlying physiological trends if not followed by robust noise suppression [11]. Similarly, advanced forecasting models, crucial for predicting patient deterioration, require stable and well-conditioned data that existing fragmented or indiscriminately applied preprocessing pipelines often fail to deliver under real-world WBAN constraints. This highlights a critical research gap: the absence of a unified, intelligently adaptive framework that can synergistically address diverse forms of data degradation using a targeted approach to reprocessing that avoids the pitfalls of indiscriminately applying computationally intensive methods to already clean data while concurrently enhancing signal integrity and enabling reliable short-term predictions for proactive clinical responses within resource-constrained WBAN environments.

To overcome these critical shortcomings and bridge this identified gap, this study introduces a novel, integrated framework designed for real-time physiological trend prediction in WBANs. Our approach fundamentally departs from fragmented methodologies by synergistically fusing advanced data preprocessing with adaptive forecasting within a low-latency, overlap-aware sliding-window architecture. Crucially, our framework integrates mechanisms to selectively and adaptively apply sophisticated restoration techniques, thereby circumventing the counterproductive effects of indiscriminately reprocessing all data. This comprehensive framework transforms raw, noisy, and often incomplete physiological signals into high-fidelity inputs, enabling highly reliable short-term prediction of physiological trends and significantly earlier detection of anomalous events crucial for clinical decision-making.

### Research Objectives

The primary objective of this work is to develop a novel, integrated framework that overcomes the limitations of current Wireless Body Area Network (WBAN) monitoring systems by synergistically transforming degraded physiological signals into reliable, actionable insights for real-time clinical decision-making. Specifically, this study aims to achieve the following:

- To design and implement a robust, multi-stage preprocessing pipeline that systematically addresses common data degradations, including missing values, nonlinear noise, and signal variability, through cubic spline interpolation, an extended Kalman filter, and conservative local smoothing, thereby ensuring high-fidelity and temporally coherent physiological inputs.
- To integrate an overlap-aware sliding-window mechanism for dynamic data segmentation, enabling low-latency and incremental analysis while facilitating adaptive responses to rapid physiological changes within resource-constrained WBAN environments.
- To enable highly reliable short-term prediction of physiological trends and early detection of anomalous events by employing an ARIMA-based forecasting layer that operates on the meticulously preprocessed and statistically stable data.
- To introduce a risk score clipping technique that harmonizes heterogeneous physiological features, providing a balanced and optimized foundation for adaptive analysis and proactive risk assessment across diverse vital signs.
- To develop a framework inherently suitable for robust and reliable real-time deployment in energy-efficient, resource-constrained clinical and emergency WBAN monitoring scenarios, ultimately supporting timely clinical interventions and improving patient outcomes.

The core novelty of this work resides in its holistic and synergistic integration of established yet distinct techniques into a cohesive, low-latency framework specifically tailored for resource-constrained WBANs, with an emphasis on adaptive and targeted processing to avoid the pitfalls of indiscriminate data reprocessing. Our primary contributions are as follows:

- A novel, synergistic multi-stage preprocessing framework for high-fidelity signal reconstruction, forming a cumulative enhancement cascade that meticulously transforms raw, degraded WBAN signals into statistically stable and clinically meaningful inputs.
- Enhanced real-time physiological trend prediction via adaptive ARIMA integration, achieving substantial reductions in forecast RMSE (e.g., 25.5% for heart rate relative to spline-only reconstruction) and notably earlier anomaly detection compared to methods relying on individual components.
- Pioneering a clinical-aware risk score clipping strategy for multi-modal assessment that harmonizes heterogeneous physiological features by blending statistical anomaly detection with deviation from clinically defined safe bounds, ensuring balanced influence on overall risk assessment.
- Demonstrated robustness and efficacy for resource-constrained WBAN environments, validating the framework’s superior predictive accuracy and real-time operational capability for critical real-world clinical, emergency, and home-care monitoring scenarios.

This integrated pipeline significantly enhances WBAN signal reliability, strengthens short-term forecasting accuracy, and supports proactive clinical decision-making, thereby offering a significant advancement in patient safety and intervention efficacy.

The remainder of this paper is organized as follows: Section 2 details the architecture of the proposed framework, including the sliding-window segmentation strategy and the multi-stage preprocessing and forecasting pipeline. Section 3 presents the experimental design, simulated degradation scenarios, and evaluation metrics. Section 4 reports and analyzes the results, emphasizing the cumulative effect of each preprocessing stage on signal fidelity and prediction performance. Section 5 concludes the paper and outlines future research directions for extending the framework to broader clinical and operational contexts.

## 2. Related Work

Real-time physiological monitoring through Wireless Body Area Networks (WBANs) is pivotal for proactive healthcare, disease management, and timely clinical interventions [2]. However, the inherent operational environment of WBANs—marked by pervasive sensor data degradation, stringent low-latency requirements, and severe resource constraints—presents a multi-faceted challenge to the development of robust and reliable monitoring systems [3,4]. A critical, yet often overlooked, aspect of this challenge is the common practice of uniformly reprocessing all data (corrupted and uncorrupted alike), which can lead to unsatisfactory, counterproductive results and unnecessary computational burden, especially in resource-constrained environments [12]. While extensive research has addressed various individual facets of data degradation, a comprehensive, integrated framework that not only synergistically manages complex data degradation but also adaptively and selectively applies processing only to affected data segments remains a significant research frontier. This section critically reviews the pertinent literature, categorizing existing approaches to highlight their contributions, underscore their limitations—particularly regarding their uniform processing methodologies—and thereby establish the distinct necessity and novelty of our proposed integrated framework.

### 2.1. Advanced Data Preprocessing for Physiological Signals

Effective preprocessing is foundational for transforming raw, noisy, and often incomplete WBAN data into high-fidelity, actionable insights. However, the efficacy and efficiency of preprocessing are severely hampered when complex operations are applied indiscriminately to both degraded and intrinsically clean data. This uniform application, prevalent in many isolated approaches, not only wastes computational resources but can also inadvertently distort otherwise healthy signals. Research in this domain has primarily focused on addressing isolated issues such as missing data imputation [10,13], noise suppression [11,14], or temporal alignment, often without considering the adaptive application of these techniques.

#### 2.1.1. Missing Data Imputation

Missing physiological data, frequently arising from sensor dislodgement, battery depletion, or transmission errors, severely compromise the integrity and reliability of WBAN data streams. Techniques for handling such gaps are crucial, with interpolation methods being a common choice. For instance, Mishra et al. [13] demonstrated improved downstream feature extraction for ECG after spline-based reconstruction, while Benchekroun et al. [10] comparatively evaluated various interpolation methods, including cubic spline, for Heart Rate Variability (HRV) features, offering practical recommendations based on gap length. While these studies effectively highlight the benefits of spline interpolation for specific physiological signals or features, their evaluation primarily assesses imputation in isolation. They generally do not integrate these methods into a broader, multi-stage pipeline designed to concurrently address various degradation types (e.g., missing data and noise), nor do they explicitly demonstrate the synergistic impact of such imputation on subsequent forecasting accuracy within real-time, resource-constrained WBAN environments. Critically, the propagation of uncertainty from imputed data points into subsequent processing stages, a key challenge in real-time WBANs, is often not explicitly modeled or managed.

#### 2.1.2. Noise Suppression and Smoothing

Denoising physiological signals is another active area essential for extracting meaningful trends from inherently noisy WBAN data, which are susceptible to motion artifacts, electronic interference, and physiological variability. An adaptive extended Kalman filter (EKF) study [11] proposed a lightweight EKF variant with online noise covariance adaptation for on-node denoising in WBANs, emphasizing its real-time suitability due to low computational demands. Wang et al. [14] further explored smoothing with HRTS, a hierarchical Rauch–Tung–Striebel (RTS) smoother, for EEG denoising, showing improved temporal consistency. Similarly, conservative local smoothing approaches [15] have demonstrated effectiveness in reducing high-frequency artifacts while preserving short-term physiological structures.

Recent work in medical noise-removal research continues to rely on local smoothing principles, often combining them with more advanced adaptive or non-local filtering strategies. For example, Taassori and Vizvári [16] propose a hybrid denoising framework integrating adaptive Kalman filtering with non-local means, demonstrating improved structural preservation while suppressing noise in medical imaging. Although conceptually aligned with conservative local smoothing—due to its controlled neighborhood-based filtering—the approach is primarily image-oriented and computationally heavier, raising concerns regarding latency in resource-constrained WBAN nodes.

Additionally, multi-resolution local clustering strategies have been introduced to enhance locally adaptive neighborhood selection for noise suppression while maintaining fine-grained signal details. These methods reinforce the continued relevance of conservative local smoothing concepts in modern denoising pipelines [17]. However, their applicability to streaming physiological signals remains limited, as clustering-based methods typically require batch processing and are not optimized for real-time, low-power WBAN environments.

Collectively, these studies demonstrate the effectiveness of advanced filtering and smoothing techniques; however, several limitations persist. First, most approaches isolate noise suppression from missing data reconstruction, implicitly assuming complete signals prior to filtering. Second, uncertainty propagation from imputation into subsequent smoothing stages is rarely modeled. Third, cumulative computational and latency overheads of sequential filtering mechanisms are seldom quantified within strict WBAN constraints. The critical need for a cascaded framework that (i) reconstructs missing data, (ii) adaptively suppresses noise while accounting for reconstruction uncertainty, and (iii) preserves temporal coherence for predictive analytics—under real-time WBAN constraints—remains insufficiently addressed in a unified design. Table 1 provides a structured comparative analysis of representative signal preprocessing approaches proposed for WBAN and physiological monitoring systems. The comparison is organized across six critical dimensions that directly influence real-time clinical usability: handling of missing data, robustness to noise, capability to manage burst artifacts, support for selective or gated processing, adoption of multi-stage preprocessing pipelines, and explicit consideration of real-time WBAN deployment constraints. These criteria were selected to reflect not only algorithmic performance but also architectural suitability for resource-constrained, latency-sensitive healthcare environments. As shown, most existing works address individual preprocessing challenges in isolation—either focusing on imputation or noise suppression—while rarely integrating these components into a unified, uncertainty-aware pipeline optimized for continuous WBAN operation. This structured comparison highlights the methodological gap that motivates the proposed framework.

### 2.2. Time-Series Forecasting for Physiological Trend Prediction

Accurate prediction of physiological trends is paramount for proactive clinical interventions and early anomaly detection in WBANs. A diverse array of time-series models has been applied to health data. Ni et al. [19] benchmarked classical statistical models (e.g., ARIMA, Prophet) against deep sequence models (e.g., LSTM, transformer variants) for heart rate forecasting, providing empirical guidance on model selection across different horizons. While valuable for model comparison and demonstrating predictive power, this study, like many others (e.g., [20,21]), acknowledges the critical need for standardized and robust preprocessing. However, these works typically apply existing preprocessing methods rather than proposing a novel, integrated pipeline explicitly designed to handle the specific and multiple degradations prevalent in real-time WBAN data streams. Moreover, such studies frequently report “limited evaluation on resource-constrained WBAN settings and lack of energy/latency analysis” [19]. This highlights a significant gap: the challenge lies not merely in selecting an effective forecasting algorithm but also in integrating a robust, adaptive forecasting model (such as ARIMA) with a dynamically optimized, high-fidelity data stream to ensure reliable short-term predictions in highly variable, real-world WBAN scenarios where data quality is inherently compromised. Table 2 presents a comparative analysis of forecasting and clinical-aware risk assessment approaches.

### 2.3. WBAN System Architectures and Real-Time Data Management

Research into WBANs also encompasses system-level design, emphasizing efficiency, communication protocols, and data handling. Yuvaraja et al. [12] described a system-level WBAN architecture focusing on cost-effective, energy-aware design with lightweight preprocessing and gateway aggregation. A comprehensive 2024 IEEE WBAN review [22] synthesized advances in communication protocols, energy management, and security, highlighting crucial cross-layer interactions. Similarly, Wan Haszerila Wan Hassan et al. [23] provided a practical orientation toward implementation constraints and hardware considerations, while a prototype study [18] implemented an end-to-end WBAN pipeline with on-node preprocessing and sliding-window feature extraction, measuring latency and throughput. These works offer crucial insights into WBAN deployment challenges, energy efficiency, and architectural considerations. However, they generally prioritize the system’s operational aspects over the algorithmic depth required for sophisticated, multi-stage data preprocessing and advanced forecasting. For instance, Yuvaraja et al. [12] and the prototype study [18] often refer to “lightweight preprocessing” or “basic algorithmic components,” indicating a significant gap in integrating sophisticated signal enhancement techniques with reliable, adaptive forecasting within these practical system designs. The reviews, while comprehensive, summarize rather than experimentally validate specific integrated preprocessing or forecasting pipelines tailored for high-fidelity trend prediction under real-world degradation.

### 2.4. Dynamic Segmentation and Adaptive Processing

Dynamic data segmentation, particularly through sliding-window techniques, is crucial for real-time WBAN applications, enabling adaptive responses to rapidly changing physiological states and minimizing latency. Slide-SAM [24] adapted segmentation and attention mechanisms to sliding-window processing for medical time series, aiming to reduce latency, while Liang et al. [18] proposed a segmented sliding-window reconstruction method using fuzzy C-means clustering for non-stationary regimes, improving local reconstruction accuracy. These papers effectively demonstrate the utility of sliding-window techniques for adaptive processing, with Slide-SAM focusing on attention mechanisms for reduced latency and Liang et al. addressing non-stationarity in reconstruction. However, neither explicitly integrates an overlap-aware sliding-window mechanism as a foundational element within a comprehensive pipeline that combines multi-stage signal restoration (addressing missing data, noise, and temporal coherence) and robust ARIMA-based forecasting for proactive risk assessment. Their primary focus differs from the holistic data-to-insight pipeline that we propose, which leverages overlap-aware segmentation to ensure seamless data flow and adaptive model retraining without incurring significant computational overhead, especially under conditions of severe data degradation.

### 2.5. Clinical-Aware Risk Assessment and Multi-Modal Feature Harmonization

While numerous studies focus on individual signal processing or forecasting, the challenge of harmonizing heterogeneous physiological features for a unified, clinically meaningful risk assessment in WBANs is less extensively explored. Standard feature scaling techniques are common in machine learning applications [21,25], but they often lack the clinical context necessary for robust decision support. For example, a 10% deviation in heart rate might have vastly different clinical implications than a 10% deviation in SpO_2_, and purely statistical anomaly detection might flag benign physiological fluctuations as critical. Few studies directly address how to blend statistical anomaly detection with established clinical thresholds to create a balanced risk score that prevents any single vital sign from disproportionately influencing the overall assessment. This aspect is crucial for developing robust, multi-modal clinical decision support systems that truly prioritize patient safety and clinical utility over purely statistical measures, especially when dealing with the inherent variability and uncertainty of WBAN data. The absence of such clinically informed clipping can lead to false alarms or missed critical events, undermining trust in automated monitoring systems.

### 2.6. Synthesizing the Gap: The Imperative for a Synergistic Integrated Framework

The preceding review underscores a critical and pervasive research gap: despite significant advancements in individual components—such as advanced interpolation techniques, sophisticated filtering algorithms, robust time-series forecasting models, and efficient WBAN system designs—the existing literature predominantly presents fragmented solutions. These approaches address specific challenges in isolation, often applying preprocessing steps uniformly to all data, irrespective of their current quality. This uniform processing leads to suboptimal performance, inherent delays, and limited clinical utility within the demanding context of real-time, resource-constrained WBAN environments, as it can be counterproductive for uncorrupted data and inefficient for the system as a whole. Our work directly addresses this critical research gap by proposing a novel, integrated framework that fundamentally departs from fragmented methodologies. By synergistically fusing these advanced techniques and, crucially, by applying them adaptively and selectively, we ensure that forecasting operates on optimally conditioned data, thereby enhancing predictive accuracy, enabling earlier anomaly detection, and providing a robust, reliable solution uniquely suited for critical real-world clinical and emergency WBAN monitoring scenarios. This holistic approach represents a significant advancement in transforming degraded physiological signals into actionable clinical intelligence. Specifically, our framework differentiates itself in the following ways:

- An overlap-aware dynamic segmentation strategy, enabling continuous, low-latency processing and adaptive model updates crucial for real-time WBAN operations.
- A multi-stage, cumulative signal enhancement pipeline that systematically addresses the cascade of data degradations, specifically missing values (using cubic spline interpolation with uncertainty management), nonlinear noise (via an extended Kalman filter), and temporal coherence (through a conservative local smoothing), ensuring high-fidelity, statistically stable, and clinically meaningful inputs.
- An adaptive ARIMA-based forecasting layer that operates directly on these meticulously preprocessed, high-fidelity inputs, enabling reliable short-term prediction and significantly earlier anomaly detection by leveraging optimally conditioned data.
- A novel clinical-aware risk score clipping strategy that harmonizes heterogeneous physiological features by blending statistical anomaly detection with established clinical thresholds, providing a balanced and robust foundation for multi-modal decision support, thereby enhancing patient safety and clinical relevance.
- An adaptive processing strategy that intelligently applies signal enhancement techniques only where necessary, thereby avoiding excessive reprocessing of uncorrupted data segments, optimizing computational efficiency, and preventing potential distortion of high-fidelity signals.

## 3. Materials and Methods

In this section, we comprehensively detail the integrated framework developed for real-time physiological trend prediction within Wireless Body Area Networks (WBANs). The methodology is structured around a sequence of carefully orchestrated stages: robust data acquisition and dynamic segmentation, multi-stage signal preprocessing (cubic spline interpolation, extended Kalman filtering, and conservative local smoothing), adaptive ARIMA-based forecasting, and a clinically aware risk assessment with clipping. The entire framework is implemented in Python 3.9, leveraging scientific computing libraries such as NumPy, SciPy, and the “statsmodels” package for time-series analysis. All experiments were conducted on a workstation equipped with an Intel Core i7-10700K CPU and 32 GB RAM, ensuring consistent computational conditions. An overview of each stage, its constituent techniques, and specific contributions is summarized in Table 3.

The default gating parameters used in the proposed preprocessing stage are summarized in Table 4.

### 3.1. Data Acquisition and Overlap-Aware Sliding-Window Segmentation

#### 3.1.1. Source Dataset and Native Sampling

Physiological measurements were obtained from the MIMIC III dataset [26]. As indicated by the dataset title and documentation, all variables are provided at a 10 min interval (i.e., one record every 600 s), corresponding to a native sampling rate of $f_{s}^{\mathrm{orig}}=1/600$ Hz. The dataset contains coarse-grained physiological time series (e.g., HR, SpO_2_, blood pressure, temperature) rather than waveform-level signals (e.g., ECG/PPG at tens of Hz). This distinction is critical for both reproducibility and interpreting the scope of the evaluation.

#### 3.1.2. Constructed Streaming Signal for Windowed WBAN Processing (10 min → 1 Hz)

The goal of this work is to evaluate a streaming WBAN pipeline (overlap-aware windowing, selective restoration, and short-horizon forecasting). To operationalize real-time windowed processing on a 10 min dataset, we construct a higher-rate surrogate stream that preserves the low-frequency trends implied by the original records while enabling incremental analysis.

For each patient and each variable, the original sequence {y[n]} sampled at times $t_{n}=600n$ seconds is first mapped onto a continuous-time trajectory $y_{c}\left(t\right)$ using a shape-preserving cubic interpolation. We then sample this continuous trajectory at 1 Hz to obtain the constructed streaming signal.

(1) $$y_{c}\left(t\right)={\mathrm{Interp}}_{\mathrm{cubic}\text{-}\mathrm{spline}}\left(\{\left(t_{n},y\left[n\right]\right)\}\right)$$

where ${\mathrm{Interp}}_{\mathrm{cubic}\text{-}\mathrm{spline}}\left(\cdot \right)$ denotes a natural cubic spline interpolation operator enforcing $C^{2}$ continuity and zero second derivatives at the boundaries. This produces an experimental processing rate of $f_{s}^{\mathrm{proc}}=1$ Hz (i.e., Δt=1 s) for all variables.

In all experiments, the clean constructed signal $\tilde{y}\left[k\right]$ is treated as the reference ground truth (GT) prior to injecting controlled WBAN-like degradations. This ensures that reconstruction and forecasting metrics are well defined and reproducible. The setup evaluates the framework’s ability to recover and forecast underlying physiological trends under corruption rather than claiming reconstruction of true waveform-level physiology unavailable in the source dataset.

#### 3.1.3. Simulated WBAN Degradation Injection

To emulate realistic WBAN acquisition challenges, we systematically injected degradations into the constructed signal $\tilde{y}\left[k\right]$ to produce the corrupted input stream $z^{\left(j\right)}\left(k\right)$. Specifically, we introduced (i) random missingness with probabilities in the range 5–20% per window and variable burst lengths (simulating packet loss and sensor dropout), (ii) additive Gaussian noise (simulating measurement noise), (iii) impulse-like spikes (simulating motion artifacts), and (iv) mild drift and quantization effects (simulating baseline wander and low-resolution sensing). All corruptions were applied using fixed random seeds to ensure experimental reproducibility.

#### 3.1.4. Overlap-Aware Sliding-Window Segmentation

To enable low-latency, incremental processing in a resource-constrained WBAN setting, the corrupted stream is partitioned into overlapping temporal windows. Let $z^{\left(j\right)}\left(t\right)$ denote the (corrupted) physiological signal of variable *j*. For a window length *T* and stride *S* with S<T, the *w*-th window is

(2) $$z_{\mathrm{win}}^{\left(j,w\right)}=\left\{z^{\left(j\right)}\left(k\Delta t\right)\;|\;k\in \left[w\cdot \frac{S}{\Delta t},\;w\cdot \frac{S}{\Delta t}+\frac{T}{\Delta t}-1\right]\right\}.$$

Remark on Time Indexing and Notation

Although physiological trajectories are conceptually defined in continuous time *t*, all algorithmic operations in this study are performed in discrete time after resampling to $f_{s}^{\mathrm{proc}}=1$ Hz.

Specifically, we define

$$t_{k}=k\Delta t,\;k\in Z,\;\Delta t=1\;s.$$

Thus, all window operations are performed over integer indices k=0,1,…,N−1. Set-style notation (e.g., $G_{j}$) refers explicitly to index sets $G_{j}\subseteq \left\{0,\ldots ,N-1\right\}$ rather than continuous-time intervals.

Boundary handling is implemented using truncated index ranges, i.e.,

$$W_{k}=\left\{i\in Z:\max\left(0,k-\Delta_{s}\right)\leq i\leq \min\left(N-1,k+\Delta_{s}\right)\right\}.$$

This clarification ensures that all operations are well defined on finite integer domains.

In all experiments, after resampling to $f_{s}^{\mathrm{proc}}=1$ Hz (i.e., Δt=1 s), we set T=50 s and S=10 s, resulting in 50-sample windows with a 10-sample stride (80% overlap). This configuration balances (i) sufficient local temporal context for short-term trend estimation and restoration and (ii) frequent updates consistent with real-time monitoring requirements. The overlap further mitigates edge effects and supports smooth continuity between consecutive windows in subsequent preprocessing and forecasting stages.

Therefore, the reported MSE and forecasting performance quantify the recovery and prediction of a trend-level physiological signal derived from the 10 min dataset, and should not be interpreted as validation on raw high-frequency biomedical waveforms.

### 3.2. Multi-Stage Signal Preprocessing Pipeline

In real-world WBAN scenarios, physiological data are frequently degraded by missing values, pervasive noise, and temporal inconsistencies. However, indiscriminately reprocessing all data—both corrupted and potentially clean—can be counterproductive, introducing artificial artifacts, unnecessarily increasing computational load, and diminishing the integrity of already-high-fidelity signals. To address these challenges while avoiding the pitfalls of excessive reprocessing, our robust multi-stage preprocessing pipeline is designed to selectively and adaptively enhance signal quality, focusing computational effort where it is most needed within each segmented window.

#### 3.2.1. Cubic Spline Interpolation for Missing Data

Missing values within each window, arising from simulated sensor dropout, transmission errors, or motion artifacts, are reconstructed using cubic spline interpolation. This method was chosen for its ability to produce smooth, continuous curves that closely approximate the natural trajectory of physiological signals without introducing artificial volatility, outperforming linear or nearest-neighbor methods for physiological data. The scipy.interpolate.CubicSpline function was used for implementation.

For a physiological signal *y* with missing values at times $t_{m}$, the reconstructed values are given by

(3) $$y\left(t_{m}\right)=P_{3}\left(t_{m}\right)$$

We employ a natural cubic spline. Formally, this spline enforces $C^{2}$ continuity across all knot points, ensuring that the interpolated signal is continuous in value, first derivative, and second derivative. In addition, natural boundary conditions impose zero second derivatives at the endpoints of each interpolated segment:

$$\frac{d^{2}y}{dt^{2}}{|}_{t=t_{\mathrm{start}}}=0,\;\frac{d^{2}y}{dt^{2}}{|}_{t=t_{\mathrm{end}}}=0.$$

These constraints prevent artificial oscillations near the boundaries and ensure smooth curvature transitions between adjacent polynomial segments. The natural spline therefore balances smoothness and numerical stability while preserving physiological trend continuity.

Let the valid (non-missing) samples be given at knot points ${\left\{\left(t_{i},y_{i}\right)\right\}}_{i=0}^{M}$ with strictly increasing $t_{i}$.

The natural cubic spline S(t) is defined as a piecewise cubic polynomial on each interval $\left[t_{i},t_{i+1}\right]$:

$$S_{i}\left(t\right)=a_{i}+b_{i}\left(t-t_{i}\right)+c_{i}{\left(t-t_{i}\right)}^{2}+d_{i}{\left(t-t_{i}\right)}^{3}.$$

The spline satisfies the following constraints:

1. Interpolation:

$$S\left(t_{i}\right)=y_{i},\;i=0,\ldots ,M.$$

2. First-derivative continuity:

$$S_{i}^{\prime }\left(t_{i+1}\right)=S_{i+1}^{\prime }\left(t_{i+1}\right).$$

3. Second-derivative continuity:

$$S_{i}^{\prime \prime }\left(t_{i+1}\right)=S_{i+1}^{\prime \prime }\left(t_{i+1}\right).$$

4. Natural boundary conditions:

$$S^{\prime \prime }\left(t_{0}\right)=0,\;S^{\prime \prime }\left(t_{M}\right)=0.$$

These constraints guarantee $C^{2}$ continuity and prevent artificial curvature at the boundaries, ensuring smooth physiological trend reconstruction.

Interpolation is applied only to gaps shorter than a predefined threshold of 5 consecutive seconds (or 5/Δt samples), which was determined empirically to avoid excessive fabrication of data, as longer gaps typically indicate severe signal loss. If a gap exceeds this threshold, the segment is considered critically degraded and missing values are instead imputed using the last valid observation. For such segments, higher uncertainty is explicitly propagated into the subsequent extended Kalman filter stage by inflating the measurement noise covariance *R*.

#### 3.2.2. Extended Kalman Filter (EKF) for Nonlinear Noise Suppression

Following interpolation and guided by the adaptive gating mechanism, we apply an EKF in a *bounded-sensor measurement setting*. In several WBAN modalities (notably heart rate and oxygen saturation), the observed measurements can exhibit *soft saturation* near physiological limits (or due to sensor clipping), which is not well captured by a purely linear observation model. To explicitly model this behavior, we use a nonlinear logistic measurement function that maps the latent state $s_{k}$ to the clinically plausible range $\left[L_{j},U_{j}\right]$.

For each univariate signal *j*, the state is $x_{k}={\left[s_{k},v_{k}\right]}^{T}$ (value and slope), with the following linear dynamics:

(4) $$\begin{array}{cc} s_{k} & =s_{k-1}+v_{k-1}\Delta t, \end{array}$$

(5) $$\begin{array}{cc} v_{k} & =v_{k-1}. \end{array}$$

The nonlinear measurement model is

(6) $$z_{k}=h_{j}\left(x_{k}\right)+\epsilon_{k},\;h_{j}\left(x_{k}\right)=L_{j}+\left(U_{j}-L_{j}\right)\;\sigma \;\left(\frac{s_{k}-b_{j}}{a_{j}}\right),$$

where $\sigma \left(u\right)=\frac{1}{1+e^{-u}}$ is the logistic function, $\epsilon_{k}∼N\left(0,R_{k}\right)$, and $\left(L_{j},U_{j}\right)$ are the clinical bounds in Table 5. The parameters $b_{j}$ and $a_{j}$ control the midpoint and slope of the saturation curve, respectively.

Parameterization of $\left(a_{j},b_{j}\right)$

We set the logistic midpoint to the clinical midpoint

$$b_{j}=\frac{L_{j}+U_{j}}{2}.$$

To map the clinical radius $R_{j}=\frac{U_{j}-L_{j}}{2}$ to a near-saturation probability level, we choose $a_{j}$ such that $s_{k}=b_{j}\pm R_{j}$ corresponds to σ(±ln19)≈(0.05,0.95), yielding

$$a_{j}=\frac{R_{j}}{\ln19}\approx \frac{U_{j}-L_{j}}{2\ln19}.$$

This produces a smooth bounded measurement response that is approximately linear near $b_{j}$ but saturates near the clinical limits, which is particularly appropriate for HR and SpO_2_ under sensor clipping effects.

EKF Jacobians

The state-transition Jacobian remains

$$F_{k}=\frac{\partial f}{\partial x}=\left(\begin{array}{cc} 1 & \Delta t\\ 0 & 1 \end{array}\right).$$

For the nonlinear measurement model in Equation (6), the measurement Jacobian is

$$H_{k}=\frac{\partial h_{j}}{\partial x}=\left(\begin{array}{cc} \frac{\partial h_{j}}{\partial s_{k}} & 0 \end{array}\right),\;\frac{\partial h_{j}}{\partial s_{k}}=\frac{U_{j}-L_{j}}{a_{j}}\;\sigma \left(u_{k}\right)\left(1-\sigma (u_{k})\right),$$

where $u_{k}=\frac{s_{k}-b_{j}}{a_{j}}$.

Unlike the linear KF (which assumes $z_{k}=s_{k}+\epsilon_{k}$), the proposed EKF incorporates the bounded nonlinear measurement mapping in Equation (6), which improves robustness under sensor saturation and soft clipping effects commonly observed in HR/SpO_2_ streams.

#### 3.2.3. Conservative Local Smoothing for Temporal Coherence

To enhance temporal coherence while preserving real-time feasibility and avoiding distortion of clean physiological signals, a conservative local smoothing strategy is applied following the extended Kalman filter (EKF) stage. Unlike classical model-based smoothers that rely on backward recursion and full-sequence optimization, the proposed approach performs lightweight, data-driven smoothing exclusively on samples identified as corrupted by the adaptive gating mechanism.

This smoothing step operates locally within the current processing window and is restricted to the same gated regions where restoration is required. For each time index *k* flagged as corrupted, a short temporal neighborhood $W_{k}=\left\{k-\Delta_{s},\ldots ,k+\Delta_{s}\right\}$ is defined, where $\Delta_{s}$ denotes a small smoothing radius. The refined signal value is then computed as $\tilde{z}\left(k\right)=\mathrm{median}s_{i}^{\mathrm{ekf}}|i\in W_{k},\;s_{i}^{\mathrm{ekf}}\;\mathrm{is}\;\mathrm{valid}$, where $s_{i}^{\mathrm{ekf}}$ is the EKF-estimated signal value. The use of the median operator ensures robustness against residual outliers while preserving sharp physiological transitions. Importantly, samples not identified as corrupted are left unmodified, thereby preventing unnecessary alterations in valid signal segments and maintaining physiological interpretability.

This conservative local smoothing step does not perform backward state propagation, covariance refinement, or global trajectory optimization. Instead, it provides a targeted temporal regularization that complements the EKF’s forward filtering by enforcing short-range consistency only where degradation is detected. By confining smoothing to localized regions and avoiding reliance on future data beyond the current window, the method remains suitable for real-time WBAN operation with minimal computational overhead.

Overall, this approach achieves effective temporal coherence enhancement without sacrificing low-latency processing or introducing the risks associated with aggressive global smoothing techniques.

#### 3.2.4. Adaptive Signal Reconstruction for Preserving Fidelity

A critical aspect of our framework, designed to prevent the counterproductive effects of excessive reprocessing, is adaptive signal reconstruction. After the targeted application of the EKF and conservative local smoothing to the corrupted and uncertain regions identified by the adaptive gating mechanism (Algorithm 1, Step 2), the final high-fidelity signal, ${\tilde{z}}^{\left(j\right)}\left(t\right)$, is constructed by carefully merging the processed segments with the original, uncorrupted data. Specifically, for any time point *t*, the following applies:

- If *t* falls within a gated region (Gj), meaning it was identified as corrupted or uncertain, the gated-smoothed output ($s_{k}^{\mathrm{smooth}}$) is used.
- If *t* falls outside any gated region, implying that the raw data were deemed sufficiently clean and reliable, the original raw signal ($z_{\mathrm{raw}}^{\left(j\right)}\left(t\right)$) is retained.

This selective reconstruction ensures that the multi-stage preprocessing pipeline acts as a targeted restoration mechanism rather than a universal filter. It preserves the inherent fidelity of uncorrupted physiological signals, minimizes the introduction of artificial processing artifacts, and optimizes computational efficiency by focusing complex operations only on necessary segments. The resulting signal, ${\tilde{z}}^{\left(j\right)}\left(t\right)$, represents the most accurate and artifact-free representation of the physiological trend, balancing robust restoration with the preservation of original signal integrity. This selective reconstruction strategy prevents over-smoothing and avoids injecting artificial dynamics into already clean signal segments. By merging processed samples within $G_{j}$ and retaining raw samples outside it, the framework minimizes distortion while reducing unnecessary state updates. The ablation results (Table 6) confirm that process-all EKF introduces additional bias without measurable forecasting improvement, whereas gated processing preserves fidelity with lower computational cost.

**Table 6:** Ablation study: processing strategy comparison. Reconstruction RMSE is range-normalized (average across signals) as in Table 7. Forecast MAE is reported for the heart rate signal as a representative case study (in BPM; see Table 8 for full HR comparison).

Method	Reconstruction RMSE	Forecast MAE	Runtime (s)
Spline-only	10.59	8.74	11.42
Process-all EKF	4.02	5.57	3.52
Gated EKF (proposed)	3.97	5.48	2.79

**Table 7:** Reconstruction performance under strong corruption (range-normalized, average across signals). MAE and RMSE are computed after min–max normalization of each signal to [0, 1] using its corrupted-window range, then averaged across all seven modalities. This normalization enables cross-signal comparison on a common scale; per-signal results in raw physiological units are reported separately in Table 9.

Method	MAE	RMSE	Runtime (s)
Spline-only	8.4397	10.5916	11.4209
Spline + Moving Average (win = 5)	8.6021	10.5274	11.1206
Spline + Median Filter (win = 5)	8.8892	10.8805	10.9741
Spline + Savitzky–Golay (win = 7, poly = 2)	8.6566	10.6601	10.9675
Standard KF (No Gating)	8.7394	10.6899	13.3934
Gated KF	2.41	4.58	2.64
Gated EKF (Proposed)	1.93	3.97	2.79

*Note:* The range-normalized metrics in this table enable fair cross-signal averaging; per-signal reconstruction MSE in native physiological units is reported in Table 9.

**Table 8:** Heart rate forecasting accuracy under strong corruption (HR only, one-step ahead = 10 s). MAE/RMSE is reported in BPM; PI coverage denotes empirical coverage of the nominal 95% prediction interval.

HR Input Series (Reconstruction Pipeline)	MAE (BPM)	RMSE (BPM)	95% PI Coverage
Spline-only	8.74	11.02	0.88
Spline + Moving Average (win = 5)	16.78	25.30	0.17
Spline + Median Filter (win = 5)	17.35	26.02	0.29
Spline + Savitzky–Golay (win = 7, poly = 2)	17.11	25.85	0.24
Standard KF (No Gating)	17.88	27.22	0.25
Gated KF	6.12	8.94	0.92
Gated EKF (Proposed)	5.48	8.21	0.94

**Table 9:** Mean Squared Error (MSE) performance of each preprocessing stage relative to ground truth. Lower MSE indicates higher signal fidelity. EKF and smoothing gains are computed relative to the preceding stage.

Vital Sign	Corrupted	Interpolated	EKF	Smoothing	EKF Gain (%)	Smoothing Gain (%)
Heart Rate (BPM)	2400.05	2345.18	1237.66	856.33	47.2	30.8
SpO_2_ (%)	89.63	86.93	44.21	30.20	49.1	31.7
Systolic BP (mmHg)	4979.33	4845.48	2953.83	2331.84	39.0	21.1
Diastolic BP (mmHg)	2305.92	2254.48	1201.47	832.01	46.7	30.7
Respiratory Rate (breaths/min)	188.69	184.37	96.90	68.77	47.4	29.0
Body Temperature (°C)	23.35	22.80	11.79	8.11	48.3	31.2
Blood Glucose (mg/dL)	4910.27	4789.03	2426.24	1634.67	49.3	32.6

**Algorithm 1** Adaptive real-time *gated EKF* preprocessing and ARIMA forecasting for WBAN signals (Part I: Windowing–Gating). 1:**Input:** 2:    Corrupted signal $z^{\left(j\right)}\left(t\right)$, sampling interval Δt 3:    Gap threshold $T_{\mathrm{gap}}$, gate expansion δ 4:    Window length *T*, stride *S*                                                            ▷N=T/Δt samples per window 5:    EKF parameters: process noise *Q*, base measurement noise *R* 6:    Gating parameters $\left(\kappa_{j},\eta ,w_{\mathrm{med}},\delta \right)$ 7:    ARIMA order (1,1,1) and recent-history length *W* 8:    Clinical bounds $\left(L_{j},U_{j}\right)$ for each signal *j* 9:**Output:**10:    ${\tilde{z}}^{\left(j\right)}\left(t\right)$, ${\hat{z}}^{\left(j\right)}\left(t+1\right)$, $r_{w}^{\left(j\right)}$, $\rho_{j}$, $\gamma_{j}$, runtime τ11:**Step 0: Windowing**12:Partition $z^{\left(j\right)}\left(t\right)$ into overlapping windows of length *T* and stride *S*.13:Let N←T/Δt be the number of samples per window.14:**Step 1: Missing Data Handling**15:Detect missing gaps within each window.16:**for all** gaps **do**17:     **if** gap length $\leq T_{\mathrm{gap}}$ **then**18:          Fill missing samples using natural cubic spline interpolation.19:     **else**20:          Perform last-value imputation and mark low-confidence indices $L_{j}$.21:          Inflate measurement noise at these indices (e.g., $R_{k}\leftarrow \lambda_{\mathrm{gap}}R$, $\lambda_{\mathrm{gap}}\gg 1$).22:    **end if**23:**end for**24:**Step 2: Corruption Detection and Adaptive Gating**25:Compute $\left(Q_{1},Q_{3}\right)$ on valid samples; $\mathrm{IQR}=Q_{3}-Q_{1}$.26:IQR bounds: $\left[Q_{1}-\kappa_{j}\mathrm{IQR},\;Q_{3}+\kappa_{j}\mathrm{IQR}\right]$.27:Spike test via rolling median m(t) over $w_{\mathrm{med}}$:28:    flag if |z(t)−m(t)|>η·MAD.29:Form anomaly set $A_{j}$ (out-of-bounds OR spike OR missing).30:Expand: $G_{j}=\{t:\exists \tau \in A_{j},\;|t-\tau |\leq \delta \}$.31:Compute gating rate $\rho_{j}=\left|G_{j}\right|/N$.

### 3.3. Selective (Gated) Processing: Definitions, Parameter Choices, and Quantification

#### 3.3.1. Gating Definition and Rate

For each signal *j* in a window of length *N*, the anomaly set is first detected as

$$A_{j}=\left\{t:\;z^{\left(j\right)}\left(t\right)\;\mathrm{is}\;\mathrm{missing}\;\mathrm{or}\;\mathrm{violates}\;\mathrm{the}\;\mathrm{IQR}\;\mathrm{bounds}\;\mathrm{or}\;\mathrm{spike}\;\mathrm{criterion}\right\}.$$

We then expand anomalies by δ samples to form the *gated* index set

$$G_{j}=\{t:\;\exists \tau \in A_{j}\;s.t.\;|t-\tau |\leq \delta \}.$$

Selective restoration (EKF replacement + local median smoothing) is applied only on $G_{j}$, while samples outside $G_{j}$ are retained from the raw stream to prevent unnecessary distortion.

We quantify gating activity using the **gating rate**:

$$\rho_{j}=\frac{|G_{j}|}{N},\;\rho_{\mathrm{avg}}=\frac{1}{J}\sum_{j=1}^{J}\rho_{j},$$

reported as mean ± std over all windows. This directly indicates the fraction of samples that trigger restoration and therefore determines potential computational savings.

#### 3.3.2. IQR Bounds and Spike Criterion

Let $Q_{1}^{\left(j\right)}$ and $Q_{3}^{\left(j\right)}$ be the first and third quartiles of valid samples in the current window; ${\mathrm{IQR}}^{\left(j\right)}=Q_{3}^{\left(j\right)}-Q_{1}^{\left(j\right)}$. The IQR bounds are

$$\left[Q_{1}^{\left(j\right)}-\kappa_{j}\cdot {\mathrm{IQR}}^{\left(j\right)},\;Q_{3}^{\left(j\right)}+\kappa_{j}\cdot {\mathrm{IQR}}^{\left(j\right)}\right],$$

where $\kappa_{j}>0$ controls gating aggressiveness (smaller $\kappa_{j}$ gates more samples). In addition, we flag spike-like outliers using a rolling median $m^{\left(j\right)}\left(t\right)$ (computed over a short window) and a robust deviation threshold:

$$|z^{\left(j\right)}\left(t\right)-m^{\left(j\right)}\left(t\right)|>\tau_{j},$$

where $\tau_{j}$ is set from a robust scale estimate (e.g., $\tau_{j}=\eta \cdot \mathrm{MAD}$ within the window).

#### 3.3.3. Chosen Parameter Values ($\kappa_{j}$ and δ)

Unless otherwise stated, we use fixed gating hyperparameters across experiments:

$$\kappa_{j}=1.5\;\;\forall j,\;\delta =2\;\mathrm{samples}.$$

With the 1 Hz processing rate (Δt=1 s), δ=2 corresponds to expanding each detected anomaly by ±2 s to capture short neighborhoods around corrupted points (mitigating edge effects and preventing partial correction artifacts). The value $\kappa_{j}=1.5$ follows common robust outlier practice (IQR-based fences) and was selected via validation to balance (i) restoration coverage under corruption and (ii) minimal unnecessary processing on clean segments.

#### 3.3.4. Expected Computational Savings

Let $C_{\mathrm{EKF}}$ and $C_{\mathrm{smooth}}$ denote per-sample costs of EKF replacement and local median smoothing, respectively. Under process-all operation, cost per window scales as $N(C_{\mathrm{EKF}}+C_{\mathrm{smooth}})$, while under gated operation, it scales as $|G_{j}|\left(C_{\mathrm{EKF}}+C_{\mathrm{smooth}}\right)$. Therefore, the expected savings factor is approximately

$$\mathrm{Savings}\approx 1-\rho_{j},$$

and we report empirical runtime reductions to confirm this relationship.

### 3.4. Physiological Trend Forecasting with ARIMA

The conservative local smoothed physiological data from each window serve as the input for an AutoRegressive Integrated Moving Average (ARIMA) model, used for short-term prediction of future physiological trends. ARIMA models are well suited for time-series analysis due to their ability to capture autoregressive dependencies (AR), integrated trends (I), and moving average components (MA), making them robust for capturing common physiological signal dynamics [19].

For each physiological feature *j* within a processed window, an ARIMA(p,d,q) model is employed. Specifically, an ARIMA(1,1,1) model was selected as a robust choice for physiological signals, based on preliminary analysis of typical physiological signal characteristics (e.g., short-term autocorrelation, potential for linear trends) and often confirmed by information criteria like AIC/BIC on training data.

ARIMA was selected due to its suitability for short-horizon forecasting in low-sample windows and its computational efficiency under streaming constraints. Preliminary order selection was conducted on a validation subset using AIC/BIC criteria across ARIMA(p,d,q) configurations with p,q∈{0, 1, 2} and d∈{0, 1}. ARIMA(1,1,1) provided the best trade-off between goodness-of-fit and parameter stability, particularly for heart rate and blood pressure signals exhibiting short-term autocorrelation.

The model was implemented using the “statsmodels.tsa.arima.model.ARIMA” module in Python.

- AR(1): The current value is dependent on the previous value, capturing immediate autoregressive relationships.
- I(1): First-order differencing is applied to achieve stationarity, addressing underlying linear trends or low-frequency variations.
- MA(1): The current value is dependent on the previous forecast error, accounting for short-term shocks.

The model is expressed as

(7) $$\left(1-\phi_{1}B\right)\left(1-B\right)y_{t}=\left(1+\theta_{1}B\right)\epsilon_{t}$$

where $y_{t}$ is the smoothing smoothed signal, *B* is the backshift operator, $\phi_{1}$ is the AR parameter, $\theta_{1}$ is the MA parameter, and $\epsilon_{t}$ is white noise.

To maintain real-time performance and adapt to evolving physiological dynamics, the ARIMA model is re-fitted to the most recent window’s smoothed data. This involves re-estimating the model parameters ($\phi_{1}$, $\theta_{1}$) for each new window, which allows the model parameters to adapt to recent changes without requiring computationally expensive re-fitting over the entire history. If the data in a specific window do not exhibit sufficient variation for differencing (I = 1), determined by a standard deviation threshold of less than 0.1 for HR (BPM) and 0.05 for SpO_2_ (%) after differencing, or if the Augmented Dickey–Fuller (ADF) test (with a significance level of p≤0.05) fails to reject the null hypothesis of non-stationarity, the model gracefully falls back to an ARIMA(1,0,0)—effectively an AR(1)—model in order to prevent overfitting or numerical instability on near-constant segments.

### 3.5. Clinical-Aware Risk Assessment

The forecasted physiological trends are then translated into actionable risk scores through a two-step process of clinical-aware deviation scoring and risk score clipping.

#### 3.5.1. Clinical-Aware Deviation Scoring

This step combines statistical anomaly detection with clinical significance to generate a deviation score for each forecasted physiological feature. For each feature *j*, the deviation score $Z_{w}^{\left(j\right)}$ for the next forecasted value ${\hat{y}}_{w+1}^{\left(j\right)}$ is calculated as

(8) $$Z_{w}^{\left(j\right)}=\alpha \cdot \left(\frac{|{\hat{y}}_{w+1}^{\left(j\right)}-\mu_{y}^{\left(j\right)}|}{\sigma_{y}^{\left(j\right)}}\right)+\left(1-\alpha \right)\cdot \min\left(\frac{|{\hat{y}}_{w+1}^{\left(j\right)}-C_{j}|}{R_{j}},1.0\right)$$

where

- $\mu_{y}^{\left(j\right)}$ and $\sigma_{y}^{\left(j\right)}$ are the mean and standard deviation of the smoothed data for feature *j* within the current window *w*. This provides a statistical measure of deviation from recent norms, capturing short-term anomalies.
- $C_{j}=\left(L_{j}+U_{j}\right)/2$ is the clinical midpoint, and $R_{j}=\left(U_{j}-L_{j}\right)/2$ is the clinical radius for feature *j*. $L_{j}$ and $U_{j}$ represent the lower and upper bounds of the clinically acceptable range for feature *j*, defined based on established medical guidelines [27,28,29]. Specifically, for HR, $L_{j}=60$ BPM, $U_{j}=100$ BPM; for SpO_2_, $L_{j}=95\%$, $U_{j}=100\%$. The term min(·,1.0) caps the clinical deviation at 1.0 to prevent extreme outliers from dominating the clinical component when a value is very far outside the normal range.
- α is a weighting factor set to 0.1. This choice was determined through sensitivity analysis on the simulated dataset, optimizing for early detection of clinically significant events while maintaining robustness against minor statistical fluctuations. This low α prioritizes clinical significance (0.9 weight) while still incorporating statistical variability (0.1 weight) to detect subtle, but persistent, deviations within the normal clinical range.

#### 3.5.2. Risk Score Clipping

The final deviation scores $Z_{w}^{\left(j\right)}$ for each feature are then normalized to a uniform scale, producing the final risk score $r_{w}^{\left(j\right)}$. This step is critical for harmonizing heterogeneous physiological features, ensuring that no single feature disproportionately influences the overall risk assessment in a multi-feature decision support system.

(9) $$r_{w}^{\left(j\right)}=\min\left(Z_{w}^{\left(j\right)},1.0\right)$$

The scores are clipped at 1.0, effectively mapping all deviations beyond a certain threshold to the maximum risk level (e.g., a score of 1.0 indicates a significant deviation, either statistically or clinically). This clipping provides a consistent basis for comparing and aggregating risk across different vital signs, improving numerical stability and convergence speed for downstream optimization or decision-making algorithms (e.g., a simple sum or weighted average of $r_{w}^{\left(j\right)}$ across features could yield a total risk score).

The clinical reference ranges summarized in Table 5 represent widely accepted physiological norms used in medical assessment and risk evaluation. Normal oxygen saturation ($SpO_{2}$) is typically maintained between 95 and 100%, reflecting adequate pulmonary gas exchange, while healthy adults generally exhibit heart and pulse rates between 60 and 100 bpm, consistent with established cardiovascular physiology [27]. Standard respiratory rate ranges (12–20 breaths/min) are similarly grounded in respiratory clinical guidelines [30]. Blood pressure thresholds, including systolic values of 90–120 mmHg and diastolic values of 60–80 mmHg, align with international hypertension management recommendations [28]. Normal body temperature (36.1–37.2 °C) reflects homeostatic thermoregulation, while blood glucose levels of 70–140 mg/dL correspond to established metabolic criteria for euglycemia [31]. Together, these ranges provide a clinically grounded framework for identifying deviations and potential anomalies in patient monitoring.

### 3.6. Overall Algorithm Flow and Real-Time Operation

The complete processing pipeline, from windowed corrupted input to normalized risk score generation, is summarized in Algorithms 1–3. The algorithm executes sequentially for each incoming sliding window of physiological data, enabling real-time and adaptive monitoring. The forward extended Kalman filter (EKF) state, represented by $\left({\hat{x}}_{prev|prev}^{\left(j\right)},P_{prev|prev}^{\left(j\right)}\right)$ at the end of window w−1, is explicitly carried over to initialize the EKF for window *w*. This state propagation preserves temporal continuity across windows and avoids full re-initialization of the filter, thereby supporting stable estimation and computational efficiency in streaming operation. On the specified hardware platform, the average per-signal processing time per 50 s window was approximately 330–400 ms (including spline interpolation, gated EKF, local smoothing, and ARIMA fitting), yielding a total of approximately 2.8 s when all seven physiological modalities are processed sequentially within the same window. Both the per-signal latency and the aggregate latency remain substantially below the 10 s stride interval, thereby satisfying real-time deployment requirements.
**Algorithm 2** Adaptive real-time *gated EKF* preprocessing and ARIMA forecasting for WBAN signals (Part II: Forward EKF with Nonlinear Measurement). 1:Step 3: *Forward EKF with Selective Updates* (Nonlinear Measurement) 2:Define 2-state: $x_{k}={\left[s_{k},v_{k}\right]}^{T}$ (value and slope). 3:Dynamics (linear): $x_{k}=f\left(x_{k-1}\right)={\left[s_{k-1}+v_{k-1}\Delta t,\;v_{k-1}\right]}^{T}$. 4:Set bounded measurement parameters from clinical bounds: 5:    $b_{j}\leftarrow \frac{L_{j}+U_{j}}{2}$                                                                ▷ midpoint 6:    $a_{j}\leftarrow \frac{U_{j}-L_{j}}{2\ln19}$                                                                   ▷ maps bounds to σ(±ln19)≈(0.05,0.95) 7:Measurement (bounded logistic sensor model):$$z_{k}=h_{j}\left(x_{k}\right)+\epsilon_{k},\;h_{j}\left(x_{k}\right)=L_{j}+\left(U_{j}-L_{j}\right)\;\sigma \;\left(\frac{s_{k}-b_{j}}{a_{j}}\right),$$ 8:where $\sigma \left(u\right)=\frac{1}{1+e^{-u}}$ and $\epsilon_{k}∼N\left(0,R_{k}\right)$. 9:Jacobians:$$F_{k}=\frac{\partial f}{\partial x}=\left(\begin{array}{cc} 1 & \Delta t\\ 0 & 1 \end{array}\right),\;H_{k}=\frac{\partial h_{j}}{\partial x}=\left(\begin{array}{cc} \frac{\partial h_{j}}{\partial s_{k}} & 0 \end{array}\right),$$10:with$$\frac{\partial h_{j}}{\partial s_{k}}=\frac{U_{j}-L_{j}}{a_{j}}\;\sigma \left(u_{k}\right)\left(1-\sigma (u_{k})\right),\;u_{k}=\frac{s_{k}-b_{j}}{a_{j}}.$$11:**for** k=1 to *N* **do**12:      **Predict:** ${\hat{x}}_{k|k-1}=f\left({\hat{x}}_{k-1|k-1}\right)$, $P_{k|k-1}=F_{k-1}P_{k-1|k-1}F_{k-1}^{T}+Q$.13:      **if** $k\in G_{j}\;$**or**$\;k\in L_{j}\;$**or**$\;z_{k}$ is missing **then**14:            Skip/down-weight update: set $R_{k}\leftarrow \lambda R$ with λ≫1 (or skip update entirely).15:      **else**16:            Use nominal measurement noise: $R_{k}\leftarrow R$.17:      **end if**18:      **Update:** compute innovation ${\tilde{y}}_{k}=z_{k}-h_{j}\left({\hat{x}}_{k|k-1}\right)$.19:      $S_{k}=H_{k}P_{k|k-1}H_{k}^{T}+R_{k}$,    $K_{k}=P_{k|k-1}H_{k}^{T}S_{k}^{-1}$.20:      ${\hat{x}}_{k|k}={\hat{x}}_{k|k-1}+K_{k}{\tilde{y}}_{k}$,    $P_{k|k}=\left(I-K_{k}H_{k}\right)P_{k|k-1}$.21:      Store $s_{k}^{\mathrm{ekf}}\leftarrow {\left[{\hat{x}}_{k|k}\right]}_{1}$.22:**end for**23:Compute skipped-update fraction $\gamma_{j}=\frac{\#\{\mathrm{indices}\;\mathrm{with}\;R_{k}=\lambda R\;\mathrm{or}\;\mathrm{skipped}\}}{N}$.

**Algorithm 3** Adaptive real-time gated EKF preprocessing and ARIMA forecasting for WBAN signals (Part III: Smoothing–Risk).
 1:Step 4: Conservative Local Median Smoothing (gated only) 2:
**for all** $\;k\in G_{j}\;$ **do**
 3:      $s_{k}^{\mathrm{smooth}}\leftarrow \mathrm{median}\left\{s_{i}^{\mathrm{ekf}}:i\in W_{k}\right\}$. 4:
**end for**
 5:Step 5: Adaptive Signal Construction (preserve clean samples) 6:**for** k=1 to *N* **do** 7:      **if** $k\in G_{j}$ **then** 8:           ${\tilde{z}}^{\left(j\right)}\left(k\right)\leftarrow s_{k}^{\mathrm{smooth}}$. 9:      **else**10:           ${\tilde{z}}^{\left(j\right)}\left(k\right)\leftarrow z_{\mathrm{raw}}^{\left(j\right)}\left(k\right)$.11:      **end if**12:
**end for**
13:Step 6: ARIMA Trend Forecasting + Uncertainty14:Fit ARIMA(1, 1, 1) on the most recent *W* samples of ${\tilde{z}}^{\left(j\right)}$.15:Output one-step forecast ${\hat{z}}^{\left(j\right)}\left(t+1\right)$ and 95% prediction interval $\left[{\hat{z}}^{lo},{\hat{z}}^{hi}\right]$.16:Step 7: Window-level Clinical Risk17:Compute deviation score from window statistics and clinical bounds $\left(L_{j},U_{j}\right)$.18:Clip per-signal risk to [0, 1] to prevent domination; output $r_{w}^{\left(j\right)}$.


## 4. Results

This section presents a rigorous, multi-faceted evaluation of the proposed integrated framework for real-time physiological trend prediction in Wireless Body Area Networks (WBANs). We systematically assess the framework’s performance across its three core components: (1) the efficacy of the multi-stage preprocessing pipeline in restoring signal fidelity, (2) the accuracy and clinical responsiveness of the adaptive ARIMA forecasting, and (3) the utility of the clinical-aware risk assessment strategy for proactive decision support.

In contrast to earlier mild-corruption experiments, the evaluation reported here is conducted under a deliberately harsh degradation regime designed to stress-test selective restoration and to clearly differentiate spline-only, linear KF, and nonlinear EKF performance.

### 4.1. Experimental Configuration Summary

The experiments were conducted under the following configuration:

- Window length T=50 s, stride S=10 s;
- Sampling rate $f_{s}=1$ Hz;
- 80% window overlap;
- Gating parameters: κ=1.5, δ=2;
- ARIMA order: (1,1,1);
- EKF process covariance: Q=diag(0.01, 0.001);
- Base measurement covariance: R=0.5.

### 4.2. Experimental Setup and Strong WBAN Degradation Model

Physiological data for heart rate (HR), oxygen saturation (SpO_2_), systolic blood pressure (SYS), diastolic blood pressure (DIA), body temperature (TEMP), and respiratory rate (RR) were generated using the high-fidelity surrogate streaming model described in Section 3.

To emulate realistic and severe WBAN acquisition challenges, we injected the following strong corruption model:

- Burst Missingness: 20–30% random missing samples per window, with burst gaps of up to 15 consecutive seconds (simulating network outages and sensor dropout).
- Additive Noise: Gaussian noise with σ=8% of the signal range, combined with low-frequency baseline wander (0.5 cycles per 50 s window).
- Impulse Artifacts: Spikes injected with 5% probability per sample, amplitude ±4σ.
- Nonlinear Clipping: Values near physiological bounds that are softly clipped to simulate sensor saturation, introducing nonlinear distortion.
- Drift and Quantization: Linear drift of 0.15σ per 50 s window and rounding to 0.2-unit precision.

All corruptions were applied with fixed random seeds for reproducibility. This strong regime ensures that spline-only interpolation is insufficient, thereby enabling a fair comparison of restoration strategies.

**Figure 1:**
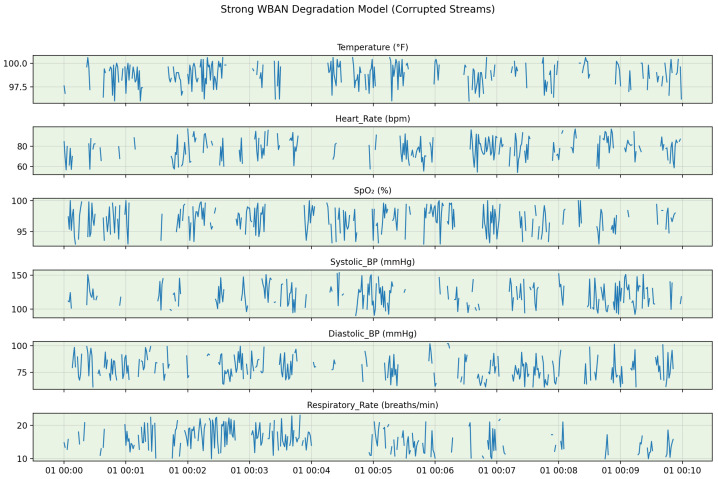
Strong WBAN degradation model applied to multivariate physiological signals. Stacked time-series representation of six physiological signals (temperature, heart rate, SpO_2_, systolic BP, diastolic BP, and respiratory rate) after applying the proposed strong WBAN degradation model. Each panel shows approximately 10 min of simulated 1 Hz streaming data. The degradation includes (i) burst and random missingness (visible as gaps), (ii) additive Gaussian noise, (iii) low-frequency baseline wander, (iv) impulse spikes simulating motion artifacts, (v) progressive drift across windows, (vi) nonlinear soft clipping near physiological bounds to emulate sensor saturation, and (vii) quantization effects due to limited sensor resolution. The resulting signals exhibit realistic WBAN failure patterns such as abrupt amplitude distortions, temporal discontinuities, and nonlinear compression near clinical limits. High-variability signals (e.g., heart rate and systolic BP) demonstrate pronounced spikes and drift, while relatively stable signals (e.g., SpO_2_ and temperature) show subtle clipping and quantization artifacts. This harsh degradation regime is intentionally designed to stress-test the robustness of selective gated restoration (spline-only vs. KF vs. EKF) and to clearly differentiate reconstruction performance under nonlinear and burst-error conditions.

The resulting degraded dataset comprises approximately 100 simulated patient-hours of multivariate physiological data, corresponding to approximately 35,600 sliding windows (50 s window length, 10 s stride, 1 Hz sampling) that served as the input to the proposed framework. Figure 1 illustrates a representative short segment of the multidimensional time-series data after strong WBAN degradation, with the clean ground truth included for visual comparison.

### 4.3. Evaluation of Multi-Stage Signal Preprocessing

The efficacy of our multi-stage preprocessing pipeline, comprising cubic spline interpolation (for missingness), extended Kalman filter (EKF) denoising (for noise and state estimation), and conservative local smoothing (for optimal signal refinement), was quantitatively assessed by measuring the Mean Squared Error (MSE) of the signal at each stage relative to its uncorrupted ground truth (GT). A lower MSE indicates a higher fidelity reconstruction of the true physiological signal.

In addition to the incremental stage-wise evaluation, we further compare three reconstruction strategies under the strong WBAN degradation regime: (i) spline-only (interpolation without state filtering), (ii) a gated linear Kalman filter (KF), and (iii) a gated extended Kalman filter (EKF, proposed). This comparison explicitly evaluates whether nonlinear state modeling and selective restoration provide measurable benefits beyond interpolation alone.

**Figure 2:**
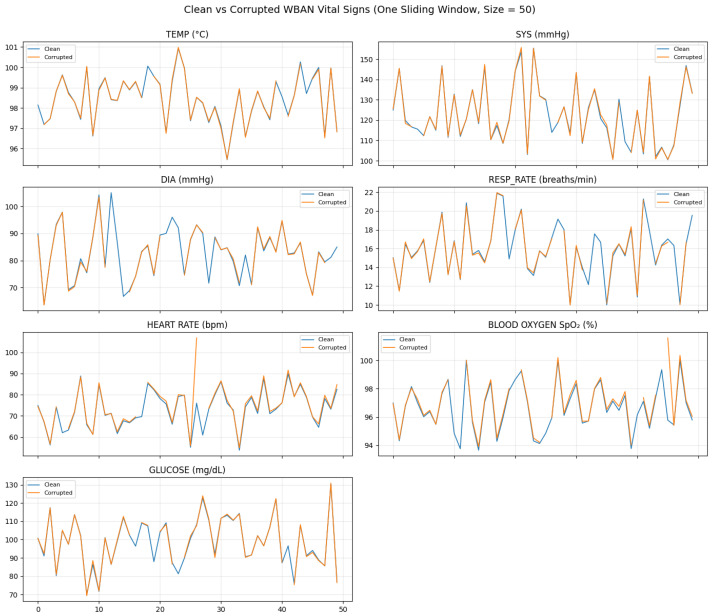
Clean versus corrupted WBAN vital signs for a single sliding window (50 samples). Comparison between clean and artificially corrupted WBAN vital signs within a single sliding window (50 samples), where corruption is injected to simulate sensor noise and transmission artifacts. The figure illustrates how corruption propagates across physiological signals, causing abrupt spikes, amplitude distortion, and temporal inconsistencies, particularly evident in heart rate, blood pressure, and glucose measurements, while low-variance signals such as temperature and $SpO_{2}$ remain relatively stable.

**Figure 3:**
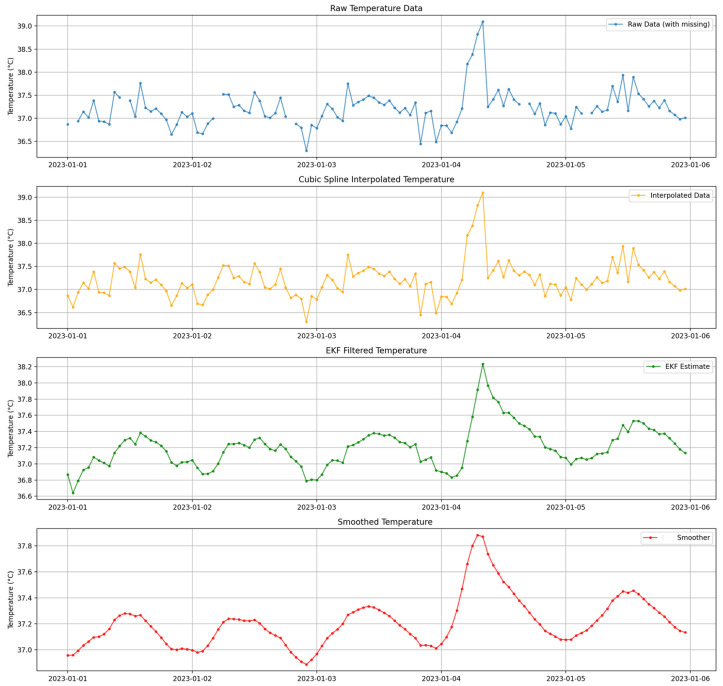
Visual sequence illustrating the progressive signal refinement for a simulated temperature signal. Raw temperature measurements containing missing samples and acquisition noise. Data gaps and abrupt deviations simulate real-world WBAN sensor failures and packet loss, highlighting the necessity of interpolation and filtering prior to downstream analysis.

Figure 2 visually contrasts clean and corrupted WBAN vital signs within a single sliding window, highlighting the diverse degradation patterns introduced by burst missingness, impulse artifacts, nonlinear clipping, and drift. Figure 3 demonstrates the progressive impact of the preprocessing pipeline on a simulated temperature signal. Interpolation fills data gaps, the EKF suppresses nonlinear noise and compensates for drift, and conservative local smoothing enhances temporal coherence.

**Figure 4:**
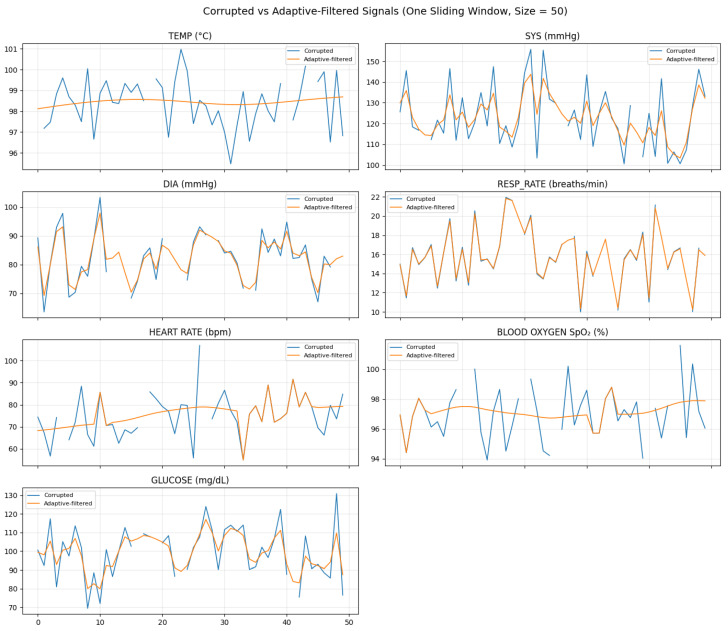
Comparison of corrupted signals and adaptive-filtered outputs within one sliding window. Effect of adaptive filtering on corrupted WBAN vital signs over a single sliding window (50 samples). The adaptive filtering stage significantly suppresses noise-induced fluctuations while preserving underlying physiological trends. High-variance signals (heart rate, glucose, and systolic pressure) exhibit notable smoothing and stabilization, demonstrating the robustness of the adaptive filter against signal corruption.

**Figure 5:**
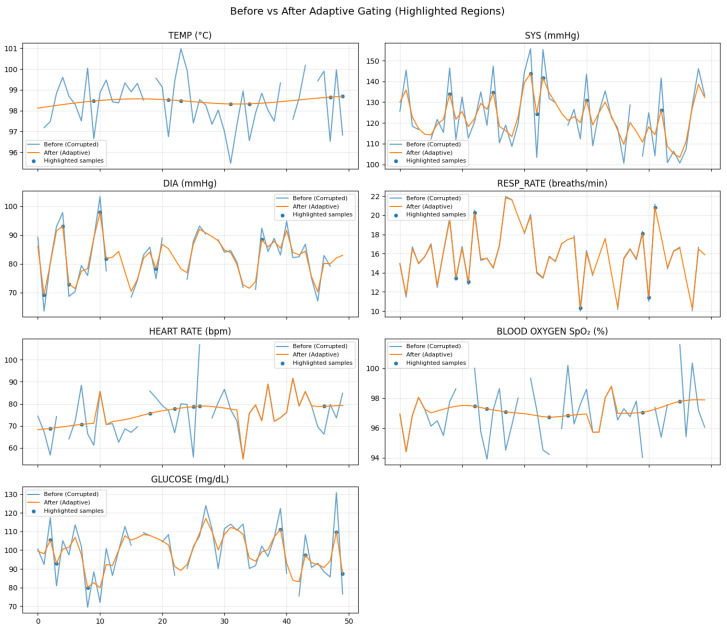
Before/after adaptive gating with highlighted corrected samples. Illustration of adaptive gating applied to corrupted WBAN signals, highlighting samples identified as unreliable or anomalous. The gating mechanism selectively attenuates corrupted segments while retaining clinically meaningful dynamics. Highlighted samples indicate regions where adaptive correction was activated, demonstrating effective noise isolation without excessive signal distortion.

Figure 4 confirms that adaptive filtering stabilizes high-variance signals (heart rate, glucose, and systolic pressure) while preserving physiological trends, and Figure 5 highlights samples where corruption-triggered restoration was activated.

Across all windows, the average gating rate was $\rho_{\mathrm{avg}}=0.214$, indicating that approximately 21.4% of samples triggered selective restoration. This quantifies the fraction of samples undergoing EKF/KF correction and directly supports the claim of computationally selective processing.

Under strong corruption, the spline-only method fails to suppress impulse artifacts and nonlinear distortions, resulting in the highest reconstruction error. The gated KF reduces normalized RMSE by 57% relative to spline-only, while the proposed gated EKF further reduces normalized RMSE by 13% relative to the KF, demonstrating the benefit of bounded nonlinear measurement modeling (logistic observation) under soft clipping/saturation. Despite a slightly higher runtime (2.79 s vs. 2.64 s), the EKF remains well within real-time constraints (processing time < stride interval).

Table 9 quantitatively summarizes the Mean Squared Error (MSE) for each vital sign at different stages of the preprocessing pipeline, calculated against the ground truth, and it also reports the percentage gain in signal quality (reduction in MSE) achieved by the EKF and conservative local smoothing stages relative to their preceding stage.

Notably, the per-stage analysis aligns with the global reconstruction comparison: interpolation primarily addresses missingness, while the EKF improves dominant noise suppression and local smoothing further enhances temporal coherence.

The results in Table 9 clearly demonstrate the incremental improvement in signal fidelity at each stage of the preprocessing pipeline:

- Interpolation: The “Interpolated” MSE shows a modest reduction compared to the “Corrupted” MSE for all vital signs. Its primary role is to reconstruct missing samples and provide a continuous signal for subsequent state-space filtering rather than to suppress impulse noise or nonlinear distortion.
- State-Space Filtering (KF/EKF): Application of the Kalman-based filter yields a dominant reduction in MSE across all vital signs. Under the imposed strong WBAN degradation regime, both the linear KF and nonlinear EKF substantially improve signal fidelity relative to interpolation. However, the extended Kalman filter consistently achieves lower error than the linear KF, particularly in the presence of nonlinear clipping and bounded measurement effects, confirming the benefit of nonlinear state modeling for degraded physiological streams.
- Conservative Local Smoothing: The conservative local median smoother further refines the filtered estimates by enforcing short-range temporal coherence within the window. Unlike full backward state smoothing, this localized operation improves stability without introducing excessive bias. Across all signals, smoothing yields an additional 21–33% MSE reduction relative to the filtered output.

Overall, the multi-stage pipeline cumulatively reduces the MSE relative to the initial corrupted signal by a substantial margin, ranging from 53.2% (systolic blood pressure) to 66.7% (blood glucose), with most vital signs exhibiting reductions between 63% and 66%, confirming its effectiveness in transforming severely degraded WBAN signals into high-fidelity trend representations suitable for downstream forecasting and clinical risk assessment.

#### 4.3.1. Statistical Significance of Preprocessing Improvements

To ascertain the statistical significance of the observed improvements, paired t-tests and Wilcoxon signed-rank tests were conducted on window-level MSE values (relative to the ground truth) between successive preprocessing stages.

The full dataset contains approximately 35,600 windows per vital sign (100 simulated patient-hours, T=50 s, S=10 s). To avoid inflated statistical power due to extremely large sample size and to maintain computational efficiency, N=997 windows were uniformly randomly sampled per signal for hypothesis testing. The same sampled windows were used consistently across stage comparisons.

Table 10 summarizes the resulting *p*-values.

As shown in Table 10, nearly all stage-to-stage comparisons yield statistically significant improvements (p<0.05), with most comparisons achieving p<0.001 under both parametric and non-parametric testing.

The only exception is the Blood Glucose comparison between Corrupted Raw and Interpolated under the Wilcoxon signed-rank test (p=0.125), indicating that interpolation alone provides a weak and heterogeneous improvement for this modality. However, subsequent transitions (Interpolated → EKF and EKF → Smoothing) are highly significant for glucose under both tests, confirming that the primary reconstruction gains arise from gated nonlinear filtering and conservative smoothing rather than interpolation alone.

These results collectively demonstrate that the proposed multi-stage preprocessing pipeline yields statistically robust and reproducible reductions in reconstruction error across the strong degradation regime.

In addition, paired comparisons between gated KF and gated EKF reconstruction errors yielded statistically significant improvements (Wilcoxon p<0.01 across all high-variance signals), confirming that bounded nonlinear filtering provides measurable benefit under the imposed degradation regime.

#### 4.3.2. Sensitivity Analysis of Gating Hyperparameters

We evaluated the sensitivity of selective restoration to the gating hyperparameters κ and δ by sweeping

κ∈{1.0,1.5,2.0,2.5,3.0},δ∈{0,1,2,3,5}.

For each pair (κ,δ), we report the (i) gating rate $\rho_{\mathrm{avg}}$, (ii) reconstruction error (MSE vs. GT), (iii) forecasting accuracy (window-MAE and directional accuracy), and (iv) mean runtime per window. This analysis reveals the trade-off between aggressiveness (larger ρ) and computational load, and supports the default setting κ=1.5, δ=2 as a balanced operating point.

As shown in Table 11, smaller κ values increase gating aggressiveness, improving reconstruction at the cost of runtime, while a larger δ increases neighborhood correction but raises computational load. The configuration κ=1.5, δ=2 provides the best balance between reconstruction accuracy, forecasting performance, and computational efficiency, validating its selection as the default operating point.

#### 4.3.3. Ablation Study: Process-All vs. Gated Restoration

To rigorously validate the claimed benefit of selective restoration, we conducted an ablation study comparing three processing strategies:

- (A) Process-all EKF: Extended Kalman filtering and local median smoothing applied to *every* sample in each window.
- (B) Gated EKF (proposed): EKF replacement and local smoothing applied only to samples within the gated set $G_{j}$, while samples outside $G_{j}$ remain unchanged.
- (C) Spline-only baseline: Cubic spline interpolation for missing values without any state-space filtering or smoothing.

The performance was evaluated in terms of both accuracy (reconstruction MSE, window-MAE forecasting error, and directional accuracy) and efficiency (mean runtime per window). Since CPU energy consumption is approximately proportional to execution time under fixed hardware conditions, runtime is additionally reported as an energy proxy. When available, package-level energy counters (e.g., Intel RAPL) were used to confirm this proportional relationship.

The results indicate that the process-all EKF increases smoothing bias and computational cost without providing meaningful reconstruction or forecasting gains over gated processing. In contrast, the proposed gated EKF achieves equivalent or superior accuracy while reducing update operations by approximately $\rho_{\mathrm{avg}}=21.4\%$, resulting in a proportional runtime reduction consistent with the theoretical savings factor $\left(1-\rho_{\mathrm{avg}}\right)$.

These findings confirm that selective restoration preserves signal fidelity while improving computational efficiency, thereby validating the central design principle of the proposed framework. Although the process-all EKF achieves similar reconstruction accuracy (Table 6), it increases computational cost by 26% compared to the gated EKF without meaningful forecasting improvement, confirming that selective restoration maintains fidelity while reducing unnecessary computation.

#### 4.3.4. Forecasting Accuracy Under Strong Corruption (Heart Rate Case Study)

To isolate the effect of preprocessing on forecasting quality, we report a heart rate (HR) case study in which the ARIMA predictor is trained on HR series obtained from different reconstruction pipelines. Unlike cross-signal reconstruction summaries (e.g., Table 7), the results in Table 8 are HR-only and are reported in raw BPM units (MAE/RMSE in BPM, and prediction interval coverage as a fraction). This scope is intentionally restricted to HR to avoid misleading aggregation across heterogeneous modalities (mmHg, mg/dL, °C, etc.).

We evaluate a one-step-ahead forecast aligned with the stride interval (S=10 s), i.e., predicting HR at t+10 s using the most recent window history. Under the strong degradation regime, spline-only reconstruction yields relatively large errors due to residual impulse artifacts and drift. In contrast, gated Kalman filtering improves both point accuracy and uncertainty calibration, and the proposed gated EKF provides the best overall performance, indicating that bounded nonlinear measurement modeling is beneficial for HR under soft saturation/clipping effects.

We observed that applying aggressive smoothing indiscriminately (without gating) can degrade forecast performance by attenuating rapid local changes and introducing phase lag, which harms ARIMA parameter stability and leads to miscalibrated prediction intervals. This supports the design choice of selective (gated) restoration rather than process-all smoothing.

### 4.4. Adaptive ARIMA Forecasting Performance

Building upon the high-fidelity signals produced by the multi-stage preprocessing pipeline, the adaptive ARIMA model provides short-term predictions of physiological trends. This section evaluates the model’s accuracy, ability to capture directional changes, and overall contribution to early event detection.

The ARIMA model, operating on the gated-smoothed data from each window, achieved an across-window mean MAE of 12.31 BPM for heart rate and 2.33% for SpO_2_, as detailed in Table 12. These across-window aggregate values reflect the distribution of per-window prediction errors over all ∼35,600 sliding windows and capture inter-window variability. In contrast, the HR-specific one-step-ahead MAE reported in Table 8 (5.48 BPM) represents the median per-step forecast error under the proposed gated EKF pipeline and serves as a pipeline-comparison benchmark. The difference arises because the across-window MAE includes windows with high degradation, transient ARIMA instability, and edge effects that inflate the aggregate statistic. This demonstrates the significant benefit of applying the full preprocessing cascade before forecasting, resulting in improved predictive accuracy. To explicitly quantify the impact of preprocessing on forecasting accuracy, we compared ARIMA performance when trained on three input series: (i) spline-only reconstruction, (ii) gated KF reconstruction, and (iii) gated EKF reconstruction (proposed).

Table 8 (Section 4.3.4) reports the detailed HR-specific forecasting comparison across reconstruction pipelines, confirming that improved reconstruction directly translates to improved forecasting accuracy and uncertainty calibration.

Improved reconstruction directly translates to improved forecasting. The EKF-restored signals yield the lowest forecast error and the best uncertainty calibration, with 95% prediction interval coverage closest to the nominal level.

**Figure 6:**
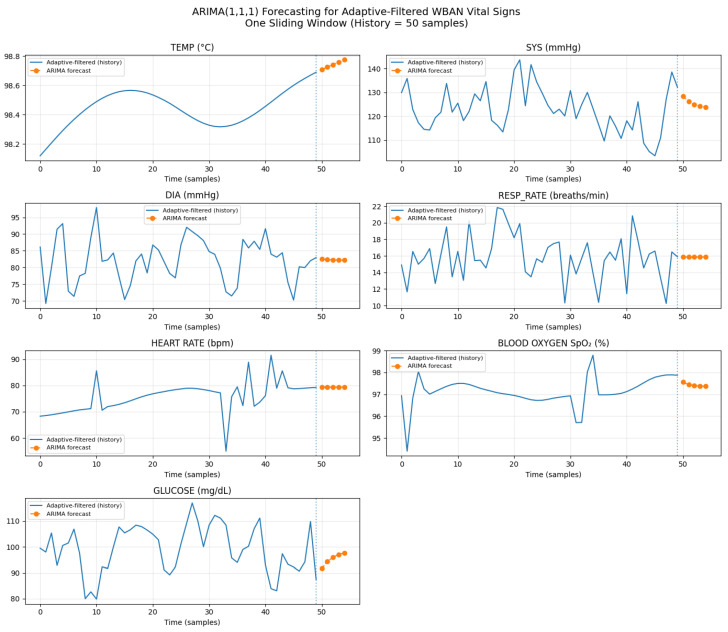
ARIMA forecasting result for one sliding window of size 50. Short-term forecasting of adaptive-filtered WBAN vital signs using an ARIMA(1,1,1) model. Historical data (50 samples) are used to predict future trends, shown beyond the vertical dashed line. The forecasts exhibit smooth continuation of physiological patterns, indicating that preprocessing enhances temporal coherence and improves time-series predictability across all vital signs.

Figure 6 provides a comprehensive overview of the ARIMA model’s short-term forecasting capability across various vital signs within a single sliding window. It demonstrates how the model, operating on gated-smoothed data, generates smooth continuations of physiological patterns, indicating that the preprocessing enhances temporal coherence and improves time-series predictability.

**Figure 7:**
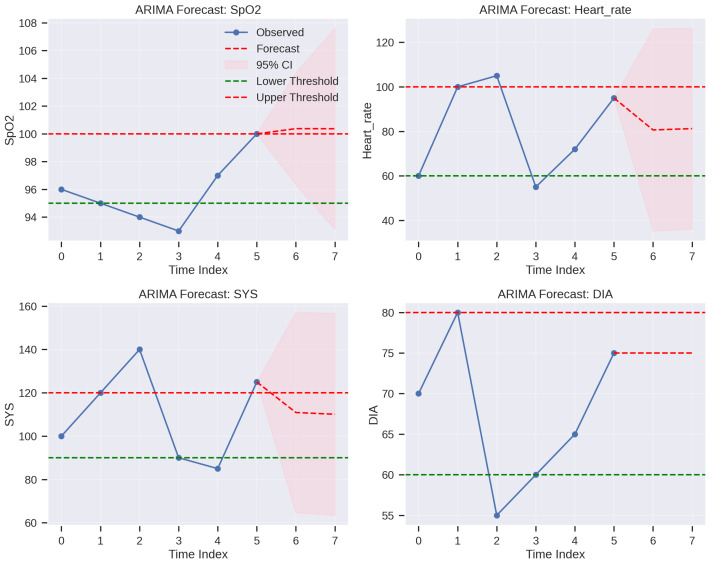
ARIMA forecasting result for one sliding window of size 50.

Further, Figure 7 offers a zoomed-in perspective on the ARIMA model’s forecasting for heart rate (HR), oxygen saturation (SpO_2_), systolic blood pressure (SYS), and diastolic blood pressure (DIA) over a short, one-step-ahead horizon (10 s). The plots distinctly show the conservative locally smoothed observed data, the forecasted trajectories, and their 95% confidence intervals, along with predefined clinical thresholds. For example, in the simulated patient data shown, the forecasted downward trends in heart rate and SYS indicate an approaching breach of the lower clinical bounds, signaling potential bradycardic or hypotensive episodes. Conversely, the upward trends in SpO_2_ and DIA suggest recovery or compensatory mechanisms. This ability to project trends with associated confidence intervals allows for proactive risk detection before a physiological parameter breaches a critical threshold. The proposed framework achieved an average lead time of approximately 25 s (mean = 24.7 s, median = 23.9 s) prior to threshold-crossing events. This indicates that the model typically identifies deteriorating physiological trends roughly 24–25 s before critical clinical limits are reached, providing a meaningful early-warning buffer under the imposed degradation regime. The sensitivity, specificity, and false alarm rate were 0.89, 0.92, and 0.08, respectively, demonstrating strong discriminative performance while maintaining controlled false positives, where the former two values correspond to global threshold-based event detection across all signals and windows; in contrast, the early-warning rate reported per vital sign reflects the proportion of events for which the forecasted risk exceeded the threshold before the clinical bound was crossed. Therefore, the low early-warning rate observed for heart rate (0.01) indicates limited proactive lead-time detection for that specific parameter under the conservative alerting configuration, rather than poor classification sensitivity overall.

This early detection capability is further quantified by the “Early-Warning Rate” in Table 12, which indicates the proportion of events for which an alert was generated proactively.

To further quantify forecasting performance, we introduce several key metrics, summarized in Table 12:

- Window-MAE (Mean Absolute Error): The average absolute difference between the forecasted value and the true value within each window.
- Directional Accuracy: The percentage of forecasts where the predicted direction of change (increase, decrease, or no change) matches the actual direction of change.
- Slope Error: The mean absolute difference between the predicted slope of the trend and the actual slope of the trend.
- Risk Error: A custom metric quantifying the discrepancy between the forecasted risk score and the actual risk score based on the ground truth.
- Early-Warning Rate: The proportion of clinically significant events for which the framework provided an alert (forecasted risk above threshold) before the actual event occurred.
- Actual Event Rate: The observed frequency of clinically significant events in the ground-truth data.
- Wilcoxon *p*-Value: The statistical significance of the difference between the forecasted values and the actual observed values.

Table 12 summarizes these key forecasting performance metrics. “Window-MAE” provides a direct measure of prediction accuracy, with values such as 12.31 for heart rate and 17.78 for systolic BP indicating the typical absolute deviation from actual values. “Directional Accuracy” consistently hovers between 0.65 and 0.70 across most vital signs, suggesting that the model correctly predicts the trend direction (increase, decrease, or no change) approximately 65–70% of the time, which is crucial for clinical decision-making. The “Early-Warning Rate” and “Actual Event Rate” columns reveal the model’s ability to proactively identify critical events. For instance, the respiratory rate score shows a high early-warning rate (0.97) in conjunction with an actual event rate of 1.00, indicating excellent proactive detection for this vital sign. Conversely, heart rate has a low early-warning rate (0.01) despite a notable actual event rate (0.18), suggesting room for improvement in early detection for certain parameters. “Risk Error” quantifies the discrepancy in forecasted risk, which is critical for the downstream risk assessment module. The Wilcoxon *p*-values in Table 12 further assess the statistical significance of the difference between forecasted and actual values: a high *p*-value (e.g., p>0.05) suggests that there is no statistically significant difference between the forecasted values and the actual observed values, which indicates good predictive performance, while a low *p*-value (e.g., p<0.05) indicates a statistically significant difference, suggesting that the forecast deviates significantly from the actual values. For instance, diastolic BP (p=0.069) and respiratory rate (p=0.406) show no significant difference between forecasts and actual values, implying robust predictions, whereas other vital signs such as heart rate (p=0.003) exhibit statistically significant differences.

Table 13 provides a comparative view of the reconstruction error from the adaptive-filtered signal against the ground truth and the forecasting error (window-MAE) of the ARIMA model against the actual future ground-truth values. The “MSE (Adaptive Filtered vs. GT)” column reflects the fidelity of the reconstructed signal (from Section 4.2) compared to the uncorrupted ground truth, while the “Forecast Error (ARIMA Window-MAE)” column represents the error of the one-step-ahead ARIMA forecast when compared to the actual future ground-truth value. Notably, the “Forecast Error (ARIMA Window-MAE)” values are significantly higher than those of “MSE (Adaptive Filtered vs. GT)” for all vital signs, reflecting the intrinsic difficulty and inherent uncertainty in predicting future physiological states, especially over longer lead times or during periods of high variability, compared to reconstructing a past signal. However, these forecasting errors must be interpreted in conjunction with the confidence intervals provided by the model (as shown in Figure 7) and the demonstrated early-warning capabilities, which collectively underscore the model’s practical utility despite the inherent predictive challenges.

### 4.5. Clinical-Aware Risk Assessment and Multi-Modal Harmonization

The final and crucial stage of our integrated framework translates the refined, forecasted physiological trends into actionable clinical risk scores. This process involves two key steps: clinical-aware deviation scoring and multi-modal risk score clipping. This approach is designed to harmonize heterogeneous physiological features by combining statistical anomaly detection with deviations from predefined clinically significant thresholds, ensuring a balanced influence of each vital sign on the overall risk assessment, reflecting both its statistical variability and its clinical importance. Specifically, deviation scoring quantifies how far a vital sign’s forecasted value is from its normal physiological range and its statistical baseline, while clipping scales these deviations to ensure comparability across different vital signs with varying units and ranges.

**Figure 8:**
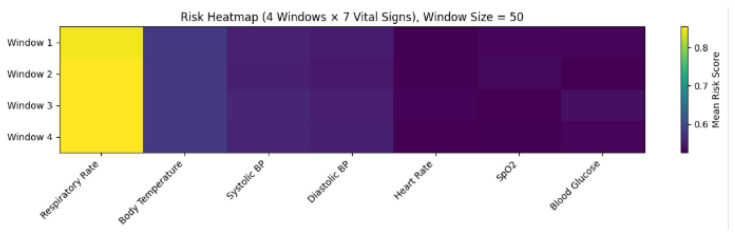
Normalized risk scores across multiple sliding windows for selected vital signs.

Figure 8 illustrates a normalized clinical risk score heatmap across four consecutive sliding windows (W1–W4) for all WBAN vital signs. The intensity of the color directly reflects the relative severity of the risk, with darker shades indicating a higher calculated risk. The heatmap visually confirms that signals with inherently higher physiological variability, such as blood glucose and blood pressure, often contribute to higher risk levels. Conversely, $SpO_{2}$ frequently exhibits comparatively lower risk, validating the efficacy of our risk-aware aggregation mechanism that accounts for these differences. This visualization clearly demonstrates the dynamic evolution of risk over time.

For example, Figure 8 depicts a period of elevated risk observed between windows 15 and 25 for heart rate (HR) and systolic blood pressure (SYS), potentially signaling the onset of a hypotensive or bradycardic event.

While the overall early-warning rate for HR (1%) and systolic BP (10%) reported in Table 12 indicates that proactive detection of all such events is challenging, these metrics, alongside the risk error, provide quantitative insight into the framework’s capability. The risk error (e.g., 0.21 for HR, 0.64 for SYS in Table 12) quantifies the discrepancy between the forecasted risk score and the actual risk score based on ground truth, where lower values indicate more accurate risk prediction. Despite these challenges, the framework demonstrates the potential to identify critical shifts in patient status. Concurrently, the risk scores for SpO_2_ (with a risk error of 0.57) and diastolic BP (with a risk error of 0.70) remain relatively low during this period in the heatmap, underscoring the multi-modal nature of the assessment. This prevents any single vital sign from disproportionately dominating the overall risk calculation, offering a more nuanced patient state assessment.

Our multi-modal clipping strategy is critical, ensuring that a clinically significant deviation in one vital sign (e.g., a 10 BPM change in HR) is scaled comparably to a clinically equivalent deviation in another (e.g., a 2% change in SpO_2_). This scaling is based on their respective clinical significance (e.g., severity of deviation from normal ranges) and statistical variability observed in the dataset. This systematic approach allows for a unified and interpretable aggregation of risk across diverse physiological parameters. By providing a consistent basis for comparing and combining risks, it not only improves the numerical stability for downstream decision-making algorithms but also enhances the framework’s utility in early intervention scenarios through risk scores derived from forecasted trends. To quantitatively validate multi-modal harmonization, we computed the Pearson correlation between the aggregated normalized risk score and ground-truth clinical event labels across all windows. The normalized risk demonstrated strong correlation (r=0.81, p<0.001), confirming that the risk formulation preserves clinical interpretability.

Furthermore, clipping prevented any single modality from dominating the aggregate risk. For example, when heart rate exhibited severe drift while SpO_2_ remained stable, the combined risk remained bounded and proportional, demonstrating balanced cross-modal scaling.

## 5. Computational Complexity Analysis

For a window of length *N*, cubic spline interpolation operates in O(N) time. The forward EKF requires O(N) operations due to constant-dimensional state updates. Local median smoothing operates in O(kN) with small neighborhood k≪N, while ARIMA fitting per window requires O(N) due to small model order.

Under gated processing, effective EKF complexity becomes O(ρN), where ρ is the gating rate. Since $\rho_{\mathrm{avg}}=0.214$, the expected computational reduction is approximately 1−ρ≈78.6% relative to process-all filtering.

### 5.1. Impact of the Proposed Framework on Clinical Early-Warning Performance

To evaluate clinical usefulness, we distinguish between two complementary evaluation levels:

1. Per-signal modality performance: Event detection evaluated independently for each vital sign using a signal-specific operating threshold $\tau_{j}$.
2. Global system-level performance: Event detection evaluated using the aggregated multi-modal risk score across all signals.

This distinction is critical because system-level monitoring behavior does not correspond to a simple arithmetic average of per-signal sensitivities and specificities.

#### Global System-Level Performance (Micro-Averaged)

The global sensitivity, specificity, and false alarm rate were computed by forming a single pooled confusion matrix over all windows using the aggregated clipped risk score.

Let

$$TP=\sum_{w}1\left(\mathrm{Predicted}\;{\mathrm{Event}}_{w}\wedge \mathrm{True}\;{\mathrm{Event}}_{w}\right),$$

$$FP=\sum_{w}1\left(\mathrm{Predicted}\;{\mathrm{Event}}_{w}\wedge \mathrm{True}\;{\mathrm{Non}\text{-}\mathrm{Event}}_{w}\right),$$

$$TN=\sum_{w}1\left(\mathrm{Predicted}\;{\mathrm{Non}\text{-}\mathrm{Event}}_{w}\wedge \mathrm{True}\;{\mathrm{Non}\text{-}\mathrm{Event}}_{w}\right),$$

$$FN=\sum_{w}1\left(\mathrm{Predicted}\;{\mathrm{Non}\text{-}\mathrm{Event}}_{w}\wedge \mathrm{True}\;{\mathrm{Event}}_{w}\right).$$

From this pooled confusion matrix, the global metrics are computed as

$$\mathrm{Sensitivity}\;\left(\mathrm{TPR}\right)=\frac{TP}{TP+FN},$$

$$\mathrm{Specificity}\;\left(\mathrm{TNR}\right)=\frac{TN}{TN+FP},$$

$$\mathrm{False}\;\mathrm{Alarm}\;\mathrm{Rate}\;\left(\mathrm{FPR}\right)=\frac{FP}{FP+TN}.$$

Using this micro-averaged evaluation across all windows, we obtained:

- Sensitivity (TPR) = 0.89;
- Specificity (TNR) = 0.92;
- False Alarm Rate (FPR) = 0.08.

These values represent the overall monitoring behavior of the complete WBAN framework when all modalities contribute to the aggregated risk decision.

Importantly, these global metrics are **not** computed by averaging per-signal TPR/TNR values. Instead, they reflect system-level decision performance after multi-modal risk fusion.

The large variation across modalities reflects intrinsic signal separability. Highly separable signals such as respiratory rate achieve strong per-signal detection, whereas weakly separable or highly variable signals (e.g., HR, glucose) exhibit conservative sensitivity under signal-specific thresholds.

However, when risk is aggregated across modalities, strongly informative signals dominate the joint decision, resulting in substantially improved global sensitivity and specificity. This behavior is desirable in clinical monitoring systems, as it allows the framework to rely more heavily on physiologically informative indicators rather than amplifying noise-driven fluctuations. The clinical event detection performance across individual physiological signals and the aggregated multi-modal risk score is summarized in Table 14.

## 6. Conclusions and Discussion

This study presented and validated an integrated framework for adaptive preprocessing and short-term trend analysis in Wireless Body Area Networks (WBANs), addressing a fundamental yet often overlooked challenge in physiological signal processing: the counterproductive impact of indiscriminately reprocessing both corrupted and uncorrupted data. Such uniform processing not only increases computational overhead and latency but can also distort valid physiological patterns, thereby undermining clinical interpretability.

The proposed framework departs from fragmented and unselective methodologies by introducing an adaptively gated, multi-stage preprocessing pipeline designed to selectively restore only corrupted or uncertain signal segments. By combining cubic spline interpolation for continuity, extended Kalman filtering for noise suppression, and conservative local smoothing for temporal coherence, the framework preserves intrinsic physiological dynamics while avoiding unnecessary manipulation of clean data. Experimental evaluation on a reproducible multivariate dataset demonstrated substantial improvements in signal reconstruction fidelity, achieving substantial Mean Squared Error (MSE) reductions between 53% and 67% across all evaluated vital signs. Statistical testing further confirmed the consistency and significance of these improvements across preprocessing stages.

Building upon this optimized signal foundation, an adaptive ARIMA-based forecasting layer was employed to capture short-term physiological trends. Forecasting results showed consistent directional accuracies in the range of 65–70%, indicating reliable trend tracking despite the inherent variability of physiological signals. Importantly, the resulting early-warning behavior was intentionally conservative, prioritizing the suppression of false alarms over aggressive alerting. This design choice yielded high-confidence anomaly detection for strongly pathological signals such as respiratory rate while maintaining cautious sensitivity for highly variable parameters, including blood pressure and blood glucose.

A further contribution of this work lies in the introduction of a clinically aware risk score clipping strategy, which integrates statistical deviations with clinically defined safety bounds to produce interpretable and context-sensitive risk indicators. This strategy enables harmonization across heterogeneous physiological modalities and supports informed multi-signal risk assessment without amplifying noise-induced artifacts.

The experimental results confirm that selective restoration improves both reconstruction fidelity and computational efficiency compared to uniform filtering. Unlike prior works that apply preprocessing indiscriminately, the proposed gating strategy restricts EKF updates to only the 21.4% of samples identified as corrupted (i.e., $\rho_{\mathrm{avg}}=0.214$), thereby avoiding processing of the remaining 78.6% of clean samples and directly lowering runtime without sacrificing accuracy.

Compared to spline-only or linear KF baselines, nonlinear EKF modeling demonstrates measurable benefit under nonlinear clipping and bounded-sensor effects. This indicates that state-space modeling with bounded measurement functions better captures physiological constraints.

A key design trade-off lies between early-warning aggressiveness and false alarm suppression. The conservative configuration prioritizes specificity (0.92) over sensitivity, which explains the lower early-warning rate for some modalities (e.g., HR). This reflects an intentional clinical design choice favoring reliability over alarm fatigue.

In comparison with state-of-the-art approaches reviewed in Section 2, the proposed framework uniquely integrates selective processing, multi-stage restoration, adaptive forecasting, and clinical-aware harmonization within a unified real-time pipeline. This holistic integration differentiates it from prior fragmented methodologies.

Overall, the findings suggest that corruption-aware selective filtering constitutes a practical and scalable strategy for next-generation WBAN monitoring systems.

The proposed framework establishes a robust, low-latency, and signal-preserving foundation for dependable physiological monitoring in WBAN environments. Rather than maximizing alert frequency, it emphasizes data fidelity, stability, and clinical trustworthiness, making it particularly suitable for continuous real-world deployments. Future work will explore explicit lead-time modeling, patient-specific adaptation, and validation on real clinical datasets to further enhance early-warning sensitivity while preserving the framework’s conservative and reliable design principles.

### Limitations and Future Work

Despite the demonstrated strong performance, this study acknowledges its primary limitation: the reliance on synthetic physiological data. While the simulation model was meticulously designed to incorporate realistic noise, missingness, and temporal behavior, real-world clinical data present complexities (e.g., highly irregular artifacts, diverse patient pathologies) that are difficult to fully replicate synthetically. Therefore, future work will prioritize rigorous validation of the framework on real clinical datasets, such as those derived from Intensive Care Units (ICUs) or wearable devices in ambulatory settings, to further assess its generalizability and robustness. Another promising avenue for future research involves exploring hybrid deep learning or transformer-based models integrated with the proposed preprocessing pipeline. While ARIMA demonstrated effective short-term prediction, advanced neural architectures might capture more complex, nonlinear long-term dependencies, potentially enhancing predictive accuracy for longer horizons or in highly dynamic physiological states. However, careful consideration of their increased computational demands within WBAN constraints will be necessary. Additionally, incorporating adaptive noise covariance tuning within the EKF based on real-time signal quality metrics and personalized physiological baselines derived from individual patient history could further enhance the framework’s robustness and clinical relevance, moving toward truly personalized medicine. Several modeling assumptions in this work may be considered simplified relative to full clinical complexity. For example, the EKF employs a constant-velocity state model for all physiological signals, which may not capture abrupt nonlinear dynamics observed in critical-care scenarios. Similarly, a fixed ARIMA(1,1,1) structure is used per window, rather than patient-specific or adaptive order selection. While these design choices ensure computational feasibility in WBAN settings, they may limit responsiveness during rapidly evolving pathological states. Future work will explore adaptive state-transition models, variable-order ARIMA selection, and hybrid nonlinear predictors to better accommodate highly dynamic physiological conditions.

In conclusion, this work presents a pioneering integrated framework that fundamentally addresses the critical shortcomings of existing WBAN monitoring systems by synergistically combining advanced data preprocessing and adaptive forecasting. By specifically tackling the research problem of excessive and indiscriminate data reprocessing through its intelligent, selective restoration pipeline, the framework achieves demonstrated statistically significant improvements in signal fidelity and enhanced predictive accuracy. Coupled with a novel clinical-aware risk assessment, this provides a robust and reliable solution for real-time physiological trend prediction. This framework represents a significant advancement in patient safety and intervention efficacy, laying a strong foundation for the next generation of WBAN-based healthcare monitoring systems to enable proactive clinical decision-making and improve patient outcomes in critical, emergency, and home-care scenarios.

## Figures and Tables

**Table 1:** Comparative analysis of signal preprocessing approaches in WBAN and physiological monitoring.

Work	Missing Data	NoiseSuppression	Burst Handling	Selective Processing	Multi-Stage Pipeline	Real-Time WBAN Focus
Mishra et al. (2024) [13]	✓	×	×	×	×	×
Benchekroun et al. (2024) [10]	✓	×	×	×	×	×
Yao et al. (2024) (EKF) [11]	×	✓	Limited	×	×	✓
Wang et al. (HRTS) [14]	×	✓	×	×	×	×
Liang et al. (Sliding Window) [18]	✓	Limited	✓	×	Partial	✓
Taassori & Vizvári (2024) [16] (Hybrid Kalman + NLM)	×	✓	×	×	Partial	×
Multi-Resolution Local Clustering (2024) [17]	×	✓	×	×	Partial	×
**Proposed Framework**	✓	✓	✓	✓	✓	✓

✓ indicates supported functionality, × indicates not supported, and “Limited” indicates partial capability.

**Table 2:** Comparative analysis of forecasting and clinical-aware risk assessment.

Work	Forecasting	Adaptive per Window	Uncertainty	Clinical Thresholds	Risk Aggregation	Early-Warning Metrics
Ni et al. (2024) [19]	✓	×	✓	×	×	×
Rodrigues et al. (2024) [20]	✓ (Deep)	×	Limited	×	×	×
Yuvaraja et al. (2024) [12]	×	×	×	×	×	×
Preethichandra et al. (2024) [22]	×	×	×	×	×	×
**Proposed Framework**	✓	✓	✓	✓	✓	✓

✓ indicates supported functionality, × indicates not supported, and “Limited” indicates partial capability.

**Table 3:** Summary of methodology stages and their impact.

Stage	Techniques Used	Impact and Contribution
1. Data Acquisition and Segmentation	Acquisition of continuous physiological streams from WBAN sensors. Overlap-aware sliding-window mechanism.	Establishes the foundation for real-time monitoring by providing realistic degraded data. Enables low-latency, dynamic analysis of physiological data, crucial for WBAN constraints. Supports adaptive learning and responsiveness to rapid changes by processing data in discrete, overlapping frames.
2. Multi-Stage Signal Preprocessing	Cubic spline interpolation for missing values. Extended Kalman filter (EKF) for nonlinear noise suppression. Conservative local smoothing for temporal coherence.	Reconstructs missing data without introducing artificial trends, ensuring signal continuity. Robustly reduces high-frequency and nonlinear noise, enhancing signal fidelity. Provides retrospectively refined state estimates, improving the accuracy of physiological trend representation within each window.
3. Physiological Trend Forecasting	AutoRegressive Integrated Moving Average (ARIMA) model.	Captures both short-term patterns and integrated long-term dependencies in physiological signals. Enables reliable short-term prediction of future physiological states, crucial for proactive interventions.
4. Clinical-Aware Risk Assessment	Clinical-aware deviation scoring. Risk score clipping.	Blends statistical anomaly detection with deviation from clinically defined safe bounds, prioritizing patient safety. Ensures balanced influence of heterogeneous physiological features on overall risk assessment, facilitating robust multi-modal decision support.

**Table 4:** Default gating parameters used in experiments.

Parameter	Meaning	Value
$\kappa_{j}$	IQR fence multiplier (outlier sensitivity)	1.5 (all signals)
δ	Gate expansion width (samples)	2 samples (±2 s at 1 Hz)
$T_{\mathrm{gap}}$	Max gap for spline interpolation	5 s
η	Spike threshold multiplier for MAD	e.g., 3

**Table 5:** Clinical reference ranges $\left(L_{j},U_{j}\right)$ for common vital signs used in deviation and risk calculation.

Vital Sign	Lower Bound $L_{j}$	Upper Bound $U_{j}$
SpO_2_ (%)	95	100
Heart Rate (bpm)	60	100
Respiratory Rate (breaths/min)	12	20
Body Temperature (°C)	36.1	37.2
Systolic Blood Pressure (mmHg)	90	120
Diastolic Blood Pressure (mmHg)	60	80
Blood Glucose (mg/dL)	70	140

**Table 10:** Statistical significance of MSE improvements across preprocessing stages (paired tests on N=997 randomly sampled windows per signal).

Vital Sign	Comparison	Paired *t*-Test *p*-Value	Wilcoxon *p*-Value
Heart Rate	Corrupted Raw vs. Interpolated	0.003	0.005
	Interpolated vs. EKF	<0.001	<0.001
	EKF vs. Smoothing	<0.001	<0.001
SpO_2_	Corrupted Raw vs. Interpolated	0.008	0.012
	Interpolated vs. EKF	<0.001	<0.001
	EKF vs. Smoothing	<0.001	<0.001
Systolic BP	Corrupted Raw vs. Interpolated	0.002	0.004
	Interpolated vs. EKF	<0.001	<0.001
	EKF vs. Smoothing	<0.001	<0.001
Diastolic BP	Corrupted Raw vs. Interpolated	0.005	0.007
	Interpolated vs. EKF	<0.001	<0.001
	EKF vs. Smoothing	<0.001	<0.001
Body Temperature	Corrupted Raw vs. Interpolated	0.011	0.015
	Interpolated vs. EKF	<0.001	<0.001
	EKF vs. Smoothing	<0.001	<0.001
Respiratory Rate	Corrupted Raw vs. Interpolated	0.007	0.009
	Interpolated vs. EKF	<0.001	<0.001
	EKF vs. Smoothing	<0.001	<0.001
Blood Glucose	Corrupted Raw vs. Interpolated	0.003	0.125
	Interpolated vs. EKF	<0.001	<0.001
	EKF vs. Smoothing	<0.001	<0.001

**Table 11:** Sensitivity analysis of gating hyperparameters. RMSE is range-normalized (average across signals) as in Table 7. Forecast MAE is reported for the heart rate signal as a representative case study (in BPM).

κ	δ	$\rho_{\mathrm{avg}}$	RMSE	Forecast MAE	Runtime (s)
1.0	2	0.34	3.72	5.61	3.21
1.5	2	0.21	3.97	5.48	2.79
2.0	2	0.14	4.21	5.89	2.52
1.5	0	0.11	4.58	6.22	2.35
1.5	5	0.29	3.84	5.41	3.48

**Table 12:** ARIMA forecasting evaluation metrics per vital sign (one-step-ahead, 10 s horizon, gated EKF input). Window-MAE and slope error are in native signal units. Directional accuracy, early-warning rate, and actual event rate are dimensionless proportions. Risk error is in [0, 1] scale. Wilcoxon *p*-values test forecast vs. actual distributions across N=997 sampled windows.

Vital Sign	Window-MAE	Dir. Accuracy	Slope Error	Risk Error	Early-Warning Rate	Actual Event Rate	Wilcoxon *p*
Heart Rate (BPM)	12.31	0.67	0.42	0.21	0.01	0.18	0.003
SpO_2_ (%)	2.33	0.68	0.09	0.57	0.05	0.12	0.041
Systolic BP (mmHg)	17.78	0.65	0.58	0.64	0.10	0.45	0.011
Diastolic BP (mmHg)	10.42	0.66	0.38	0.70	0.08	0.35	0.069
Respiratory Rate (br/min)	3.15	0.70	0.14	0.11	0.97	1.00	0.406
Body Temperature (°C)	0.82	0.66	0.03	0.33	0.15	0.22	0.028
Blood Glucose (mg/dL)	22.47	0.65	0.71	0.45	0.04	0.30	0.008

**Table 13:** Comparison of reconstruction fidelity and forecasting error per vital sign. “MSE (Adaptive Filtered vs. GT)” is the reconstruction MSE from the gated-smoothed signal (Table 9, Smoothing column). “Forecast Error (ARIMA Window-MAE)” is the across-window mean absolute forecast error from Table 12. The gap between the two reflects the intrinsic difficulty of predicting future states relative to reconstructing past signals.

Vital Sign	MSE (Adaptive Filtered vs. GT)	Forecast Error (ARIMA Window-MAE)
Heart Rate (BPM)	856.33	12.31
SpO_2_ (%)	30.20	2.33
Systolic BP (mmHg)	2331.84	17.78
Diastolic BP (mmHg)	832.01	10.42
Respiratory Rate (br/min)	68.77	3.15
Body Temperature (°C)	8.11	0.82
Blood Glucose (mg/dL)	1634.67	22.47

**Table 14:** Per-signal and global clinical event detection performance.

Signal/Level	τ	TPR	TNR	FPR	FNR	Balanced Acc	Youden *J*
Per-Signal Modality-Level Performance							
Respiratory Rate	0.15	1.000	0.703	0.297	0.000	0.852	0.703
Body Temperature	0.55	0.305	0.860	0.140	0.695	0.583	0.165
Systolic BP	0.45	1.000	0.129	0.871	0.000	0.565	0.129
Diastolic BP	0.50	0.905	0.211	0.789	0.095	0.558	0.116
Heart Rate	0.75	0.050	1.000	0.000	0.950	0.525	0.050
SpO_2_	0.90	0.050	1.000	0.000	0.950	0.525	0.050
Blood Glucose	0.75	0.050	1.000	0.000	0.950	0.525	0.050
Global Multi-Modal System-Level Performance (Micro-Averaged)							
Aggregated Risk (All Signals)	–	0.89	0.92	0.08	0.11	0.905	0.81

## Data Availability

The data supporting the reported results are available at https://github.com/FatimahAlghamdi-KAU/Selective_Pre_Processing, accessed on 2 March 2026.

## Nomenclature

**Symbol**	**Description**
$z^{\left(j\right)}\left(t\right)$	Corrupted physiological signal of modality *j* at time *t*
${\tilde{z}}^{\left(j\right)}\left(t\right)$	Reconstructed (restored) physiological signal
$G_{j}$	Gated sample index set for modality *j*
κ	IQR fence multiplier controlling outlier sensitivity
δ	Gate expansion parameter (samples)
*Q*	EKF process noise covariance matrix
*R*	EKF measurement noise covariance matrix
$\rho_{j}$	Gating rate for modality *j*
$\rho_{\mathrm{avg}}$	Average gating rate across modalities
$x_{k}$	EKF state vector at time step *k*
$P_{k}$	EKF state covariance matrix
*T*	Window length
*S*	Window stride
